# Highly Sensitive Spatial Host‐Microbiome Transcriptomics in FFPE Tissues via Iterative Hydrogel Expansion

**DOI:** 10.1002/advs.77219

**Published:** 2026-08-17

**Authors:** Shunji Zhang, Mengyuan Wang, Jiaye Chen, Yuyi Zhu, Bin Zhu, Ziye Xu, Yuan Liao, Yongcheng Wang

**Affiliations:** ^1^ Department of Laboratory Medicine of The First Affiliated Hospital & Liangzhu Laboratory Zhejiang University School of Medicine Zhejiang University Hangzhou China; ^2^ College of Biomedical Engineering and Instrument Science Zhejiang University Hangzhou China; ^3^ M20 Genomics Hangzhou China

**Keywords:** expansion, FFPE, random primer, spatial transcriptomics

## Abstract

Spatial transcriptomics (ST) of archival formalin‐fixed paraffin‐embedded (FFPE) tissues is fundamentally limited by a trade‐off between spatial resolution and transcriptomic sensitivity due to severe molecular crowding and RNA degradation. Here, we present Ex‐spRandom, a highly sensitive platform that overcomes this limitation by directly converting diverse, fragmented RNA biotypes into stably anchored cDNA through the integration of random‐primed in situ cDNA synthesis chemistry with interpenetrating polymer network (IPN)‐based tissue expansion chemistry. This physicochemical synergy physically decrowds the dense FFPE matrix, directly converting highly fragmented host and microbial RNAs into stably anchored cDNA. Consequently, Ex‐spRandom improves spatial signal confinement while enabling the detection of nearly 10,000 median genes per 50‐µm spatial bin. Leveraging this synergistic high resolution and sensitivity, the platform captures over 44,000 unique host genes, enabling the precise delineation of continuous neurodevelopmental trajectories in the complex embryonic eye. Furthermore, Ex‐spRandom achieves simultaneous, spatial profiling of host epithelial architecture and microbial transcript signals in the colon, detecting over 150,000 unique microbial genes. Ultimately, this scalable platform establishes a framework for sensitive, cross‐kingdom spatial profiling in archival FFPE tissues.

## Graphical abstract

Ex‐spRandom is a spatial transcriptomics platform that synergizes random‐primed chemistry with iterative hydrogel expansion. By physically decrowding the dense FFPE matrix, this scalable technology shatters the traditional resolution‐sensitivity barrier. It enables simultaneous, high‐resolution spatial mapping of host epithelial architectures and resident microbiomes, unlocking ultra‐sensitive cross‐kingdom molecular profiling for archival tissue analysis.

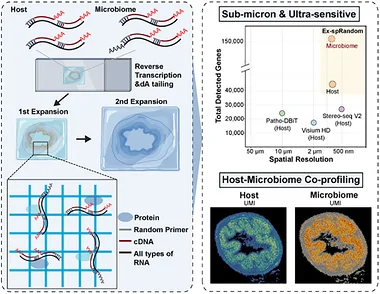

## 1 Introduction

Spatial transcriptomics (ST) offers unprecedented insights into cellular microenvironments, yet unlocking the immense molecular information within large‐scale clinical archives remains a critical challenge [1, 2, 3]. The vast majority of these invaluable biospecimens are preserved as formalin‐fixed paraffin‐embedded (FFPE) tissues [4, 5]. The highly degraded nucleic acids and densely cross‐linked macromolecular networks inherent to FFPE matrices pose severe physicochemical barriers, limiting the simultaneous attainment of high transcriptomic sensitivity and high spatial resolution [6, 7].

Current spatial transcriptomic approaches address this resolution–sensitivity trade‐off through different technical strategies. Existing unbiased spatially barcoded systems face a severe physical‐molecular dilemma: conventional macro‐scale arrays are constrained by physical spatial limits (typically 10–50 µm) [8, 9, 10], whereas ultra‐high‐density nano‐arrays achieve sub‐cellular resolution at the cost of reduced RNA capture efficiency [11, 12, 13, 14]. Alternatively, high‐resolution targeted in situ approaches circumvent these capture limitations by using predefined finite gene panels [15, 16, 17]. However, this inherent bias obscures broad‐spectrum exploratory signals, failing to capture crucial non‐coding RNAs and tissue‐resident microbial transcripts [18, 19]. This technological impasse is further exacerbated by the inherent defects of the FFPE matrix itself. The densely cross‐linked protein network introduces profound spatial steric hindrance, which severely restricts reagent penetration and transcript accessibility [6, 7]. Consequently, engineering a unified platform that simultaneously improves spatial resolution and whole‐transcriptome sensitivity in degraded FFPE tissues remains a major technical challenge.

Several studies have combined tissue expansion with transcriptomic readouts, including ExSeq [20], expansion‐MERFISH [21], EASI‐FISH [22], and expansion spatial transcriptomics [23]. However, these methods have mainly been demonstrated in non‐FFPE samples, and their applicability to fragmented FFPE RNA and simultaneous host–microbial transcript detection remains limited. To overcome these interconnected physical and molecular bottlenecks, we developed Ex‐spRandom, an optimized spatial transcriptomics platform tailored for highly degraded FFPE tissues. Building upon a random‐primed in situ cDNA synthesis chemistry [24, 25] and an interpenetrating polymer network (IPN)‐based tissue expansion chemistry [20, 26], our approach directly converts diverse, fragmented RNA biotypes into stably anchored cDNA. Subsequent physical de‐crowding effectively alleviates the spatial steric hindrance inherent to the FFPE matrix. Synergizing this unbiased capture chemistry with physical expansion improves the effective spatial resolution of accessible macro‐scale arrays. Consequently, under the standardized 50‐µm binning and downstream reanalysis framework, Ex‐spRandom showed the highest median gene detection among the analyzed mouse brain datasets, with nearly 10,000 genes detected per spatial bin [10, 11]. This demonstrates the platform's capacity to capture over 44,000 host genes and over 150,000 microbial genes across diverse complex microenvironments. This dual leap empowered us to unlock deeper dimensions of spatial analysis, successfully unmasking seamless developmental trajectories in the highly dense embryonic eye and achieving the simultaneous, high‐fidelity spatial mapping of host epithelial architecture alongside resident microbiomes within the colon. Ultimately, by capturing these previously inaccessible low‐abundance and cross‐kingdom signals, Ex‐spRandom establishes a robust, highly scalable framework for deciphering complex structural and functional microenvironments in FFPE tissues.

## 2 Workflow of the Ex‐spRandom Platform

To achieve high effective spatial resolution and high transcriptomic sensitivity in highly degraded formalin‐fixed paraffin‐embedded (FFPE) tissues, we developed Ex‐spRandom, a platform that builds upon tissue expansion chemistry integrated with random‐primed in situ transcript capture chemistry [24, 25] (Figure 1a). This strategy addresses the long‐standing trade‐off between spatial resolution and transcriptomic sensitivity in spatial transcriptomics. The workflow begins with in situ de‐crosslinking and permeabilization of FFPE sections on adhesive slides to relax the densely cross‐linked protein matrix and restore molecular accessibility. This pretreatment enables efficient diffusion of enzymatic reagents into the tissue while preserving the native spatial architecture. Subsequently, Ex‐spRandom employs an unbiased reverse transcription (RT) strategy based on random primers to initiate transcript capture directly within the tissue. This critical molecular design reduces dependence on conventional poly(A)‐dependent limitations, ensuring highly efficient in situ conversion of fragmented host mRNAs, non‐coding RNAs, and exogenous bacterial transcripts into cDNA. As a result, diverse RNA species present in degraded FFPE samples can be captured with minimal bias. Following reverse transcription, the synthesized cDNA molecules undergo in situ poly‐dA tailing, which appends capture adapters to their 3′ ends and enables stable downstream anchoring. To facilitate non‐destructive tissue release, a thermomechanical freeze–thaw step (−80°C–25°C) is introduced to weaken the adhesion between the tissue and the slide. Subsequently, a rationally formulated monomer solution is then infused into the tissue and polymerized to form a dense interpenetrating polymer network (IPN), physically entrapping the poly‐dA‐tailed cDNA molecules within the hydrogel matrix. The resulting tissue–hydrogel composite subsequently undergoes isotropic expansion in 1× PBS, which physically decrowds the dense biological matrix while maintaining spatial registration of the anchored cDNA and reducing lateral diffusion. For biological architectures requiring enhanced spatial precision, the system supports iterative physical expansion through secondary hydrogel re‐embedding, enabling further amplification of effective spatial resolution [27]. Finally, the expanded hydrogel is transferred onto a spatial barcode array through a streamlined, equipment‐free protocol, allowing cost‐effective spatial transcriptomic sequencing. The quantitative advantage of this integrated physicochemical strategy is illustrated in the resolution–sensitivity phase space (Figure 1b). By translating physical tissue expansion into spatial decoding power, Ex‐spRandom substantially shifts the achievable sensitivity at high spatial resolution. Compared with state‐of‐the‐art spatial platforms—such as Visium HD, Patho‐DBiT [10], and Stereo‐seq V2 [11]—Ex‐spRandom simultaneously achieves high effective spatial resolution while maintaining high transcriptomic breadth in FFPE tissues under the analyzed comparison framework (Figure 1b). It captures over 44,000 host genes per FFPE sample and detects more than 150,000 microbial genes, supporting sensitive host–microbiome spatial profiling (Figure 1b).

**FIGURE 1:**
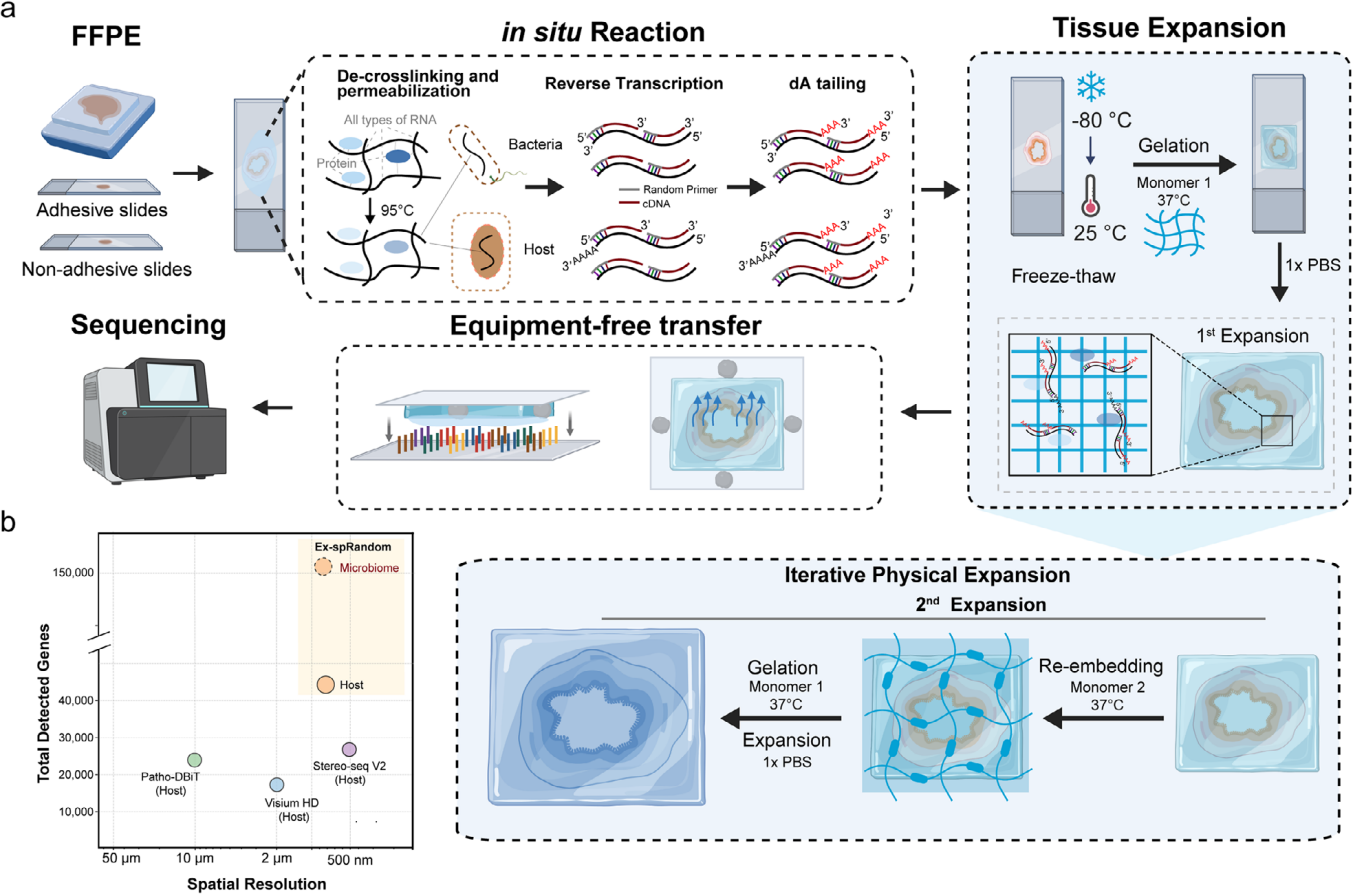
Schematic overview and technology comparison of the Ex‐spRandom platform. (a) Workflow of Ex‐spRandom for FFPE tissues. The protocol includes in situ de‐crosslinking and permeabilization, followed by random‐primed reverse transcription and poly‐dA tailing. The tissue then undergoes freeze–thaw detachment and hydrogel gelation for expansion, with optional iterative re‐embedding for multiple expansion rounds. The expanded tissue gel is subsequently transferred onto barcode arrays for sequencing. (b) Quantitative benchmarking of joint spatial resolution and transcriptomic sensitivity, comparing Ex‐spRandom with state‐of‐the‐art platforms (Visium HD, Patho‐DBiT [10], and Stereo‐seq V2 [11]).

## 3 Validation of Physical Isotropy and Molecular Integration in the Ex‐spRandom Workflow

To determine whether hydrogel expansion can be reliably integrated into random‐primed whole‐transcriptome sequencing, we systematically validated the Ex‐spRandom workflow, progressing from macroscopic physical properties to molecular cDNA output. We first evaluated the macroscopic swelling capacity of five distinct hydrogel formulations (A to E) (Table S1 and Figure S1). These formulations were rationally designed by modulating the backbone ratios of sodium acrylate (SA) and acrylamide (AA), tuning the density of the crosslinker N,N'‐methylenebisacrylamide (BIS) or substituting it with N,N'‐ethylenebisacrylamide (EBIS), and selectively incorporating N,N‐dimethylacrylamide (DMAA) to optimize the polymer chain. Utilizing an 8‐mm pre‐expansion reference template, we found that the linear expansion factors exhibited a monotonic increase from 2.16 to 3.62 in a gel‐only context (Figure 2a,b). However, applying this to standard FFPE sections resulted in incomplete tissue release from adhesive slides during PBS‐buffered expansion (Figure 2c, left). By introducing a −80°C pre‐incubation step, we successfully leveraged thermomechanical phase transition to decouple the tissue‐slide interface, ensuring smooth and complete detachment (Figure 2c, right). Multi‐angle geometric measurements across diverse FFPE tissues—including embryonic head regions, olfactory bulb, intestine, and brain sections (Figure 2d,e)—yielded mean expansion factors of ∼3.4 on non‐adhesive slides and ∼3.1 on adhesive slides. This indicated a mild, predictable physical constraint imposed by the tissue matrix and surface coating relative to pure hydrogels. Beyond macroscopic scaling, preserving absolute spatial fidelity during expansion is paramount. DAPI staining of FFPE mouse embryo head sections supported isotropic structural preservation, evidenced by a ∼3.6‐fold uniform scaling with spatial alignment in pre‐ and post‐expansion overlay images (Figure 2f). Furthermore, within structurally heterogeneous regions such as the FFPE mouse hippocampus, we quantified isotropy by calculating inter‐nuclear distance ratios (e.g., L1'/L1 and L2'/L2) between matched individual nuclei before and after expansion [28]. These micro‐scale geometric measurements, strongly corroborated by rigid image registration (Figure 2g,h), confirmed that the complex tissue architecture expands uniformly without obvious localized structural distortion.

**FIGURE 2:**
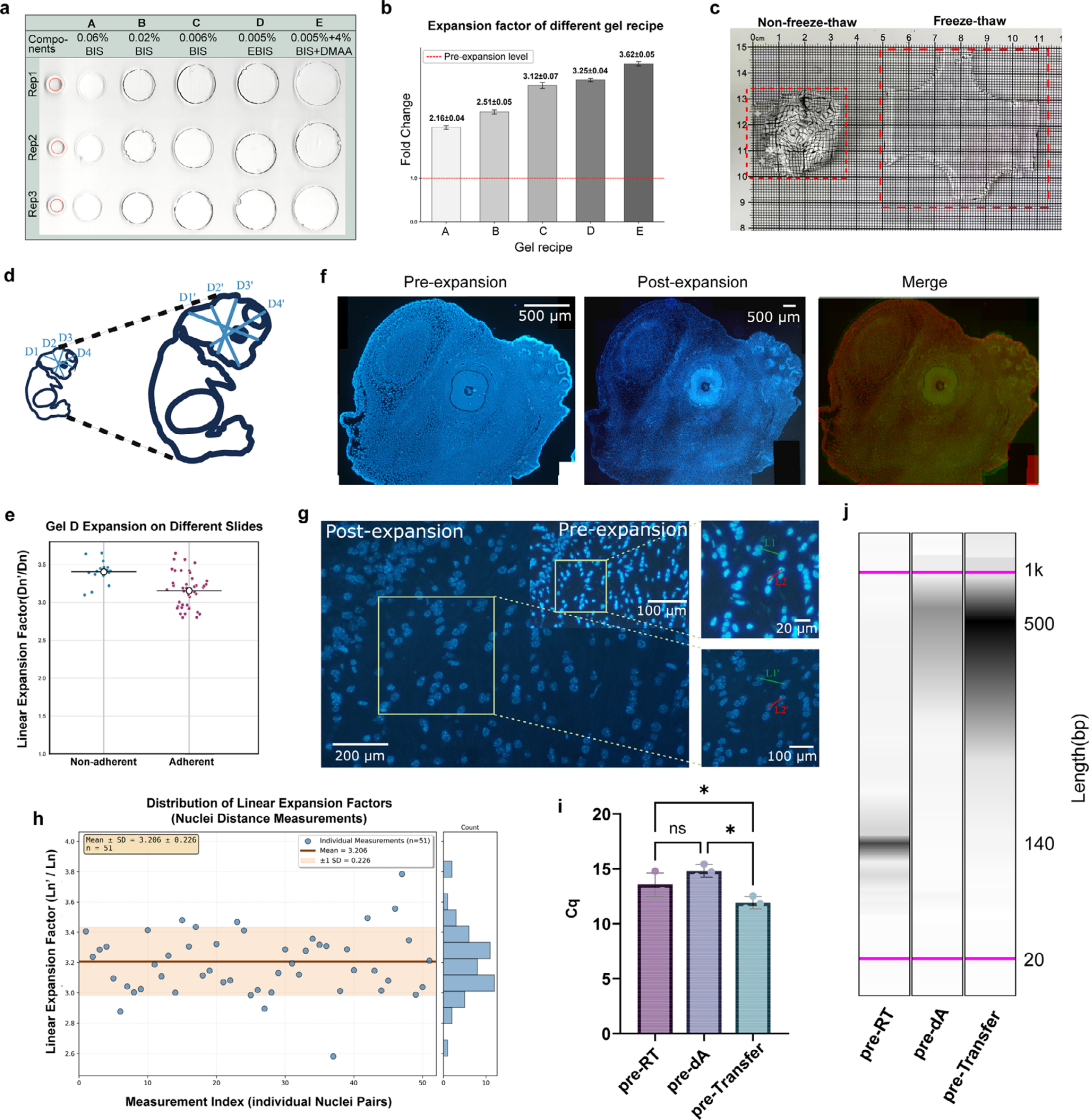
Technical validation of Ex‐spRandom. (a) Macroscopic swelling assay of five hydrogel formulations (A–E) without tissue; the first‐column red circle indicates the pre‐expansion diameter (8 mm), and each formulation was tested in triplicate. (b) Linear expansion factors from panel A (post/pre) for formulations A–E. (c) FFPE tissue release test on adhesive slides, showing failed detachment without freezing (left) and successful release after a ‐80C freezing step (right). (d) Schematic of multi‐angle isotropy measurement in an FFPE mouse embryo head section using matched pre/post positions across four directions. (e) Expansion quantification across FFPE mouse olfactory bulb, embryo, intestine, and brain based on panel D. (f) DAPI images of an FFPE mouse embryo head section before and after expansion plus red/green merged registration. (g) High‐magnification DAPI comparison in FFPE mouse hippocampus before and after expansion, showing representative inter‐nuclear distance measurements. (h) Quantification of panel G. (i) qPCR cycle comparison across three workflow positions for introducing expansion into spRandom to generate Ex‐spRandom in FFPE tissues (pre‐RT, pre‐dA, pre‐Transfer); statistical significance was assessed using Tukey's multiple comparisons test (*, *p* < 0.05; ns, not significant). (j) Qsep quality assessment of transferred products from panel I.

Finally, we sought to pinpoint the optimal integration point for molecular capture within the spRandom pipeline. We systematically compared cDNA yield and library quality across three workflow configurations: integrating expansion prior to reverse transcription (pre‐RT), prior to poly‐dA tailing (pre‐dA), and after complete in situ synthesis but before array transfer (pre‐Transfer). Quantitative PCR benchmarking showed that the pre‐Transfer configuration had a favorable Cq profile among the tested conditions, although not all pairwise comparisons were statistically significant (Figure 2i and Figure S2a). Therefore, the workflow choice was not based solely on qPCR. Capillary electrophoresis (Qsep) profiling further supported the selection of pre‐Transfer: whereas the pre‐RT strategy yielded fragmented, short amplicons, and the pre‐dA strategy suffered from severely reduced concentrations, the pre‐Transfer protocol maintained the expected fragment‐size distribution and higher molecular yield (Figure 2j and Figure S2b). Collectively, these physical and biochemical optimizations establish pre‐Transfer as the definitive Ex‐spRandom configuration, yielding a highly efficient, end‐to‐end workflow that preserves both sub‐cellular geometry and whole‐transcriptome integrity.

## 4 Enhanced Whole‐Transcriptome Capture Efficiency of Ex‐spRandom in FFPE Tissues

To investigate whether tissue expansion could counteract RNA degradation and enhance transcriptomic sensitivity in FFPE samples, we implemented Ex‐spRandom on the mouse hippocampus. Despite utilizing a 50‐µm macro‐scale array, Ex‐spRandom enabled cell‐type deconvolution of diverse cell populations via RCTD [29], uncovering refined architectural details that were previously masked. These spatial patterns were consistent with the classical anatomical landmarks of the hippocampus and showed concordance with DAPI‐stained histological features (Figure 3a). Notably, within core structures such as the Cornu Ammonis (CA1, CA3) and dentate gyrus (DG), Ex‐spRandom exhibited sharper cellular boundaries and reduced spatial smearing compared to standard spRandom [24] (Figure 3b). Notably, quantitative assessment of spot composition confirmed that this enhanced resolution dramatically shifted the distribution of cellular mixtures; the median maximum cell‐type proportion per spot increased significantly, accompanied by a nearly ten‐fold surge in the fraction of high‐purity spots compared to unexpanded arrays (Figure S3a). To benchmark transcriptomic sensitivity in FFPE mouse hippocampus, we compared Ex‐spRandom against standard spRandom and public spatial datasets. To minimize potential biases introduced by tissue region, spatial aggregation strategy, and dataset processing differences, all datasets were evaluated using a standardized 50‐µm final analysis bin. When evaluated at an equivalent 50‐µm spatial resolution, Ex‐spRandom consistently achieved the highest median gene and UMI capture rates. Notably, it achieved a median detection of 10,007 genes per spatial bin, indicating enhanced transcriptomic detection capacity across the analyzed datasets within this standardized benchmarking framework (Figure 3c and Figure S3b and Table S2). Under the standardized analysis framework, Ex‐spRandom detected an high number of 44,698 unique host genes, compared with other unbiased whole‐transcriptome methods—including spRandom [24] (31,857), Stereo‐seq V2 [11] (27,553), and Patho‐DBiT [10] (23,807)—as well as targeted probe‐based platforms like Visium HD (18,368), Xenium [16], and MERFISH [15] (Figure 3d and Figure S3c). Beyond protein‐coding genes, Ex‐spRandom also showed improved detection of non‐coding RNA species. It recovered an increased number of lncRNAs (9,193), miRNAs (714), snRNAs (807), and snoRNAs (564) across the evaluated datasets (Figure 3e). Ex‐spRandom further recovered additional transcripts, including regulatory non‐coding RNAs and pseudogenes, that were not detected in standard array‐based datasets (Figure S3d,e). Ex‐spRandom demonstrated technical reproducibility across adjacent sections, with high correlation in both global gene expression and the profiles of key transcription factors (R^2^ = 0.99). Furthermore, the spatial expression patterns captured by Ex‐spRandom were consistent with those from conventional spRandom and MERFISH datasets, supporting the reliability of its gene detection and spatial mapping performance (Figure 3f and Figure S3f).

**FIGURE 3:**
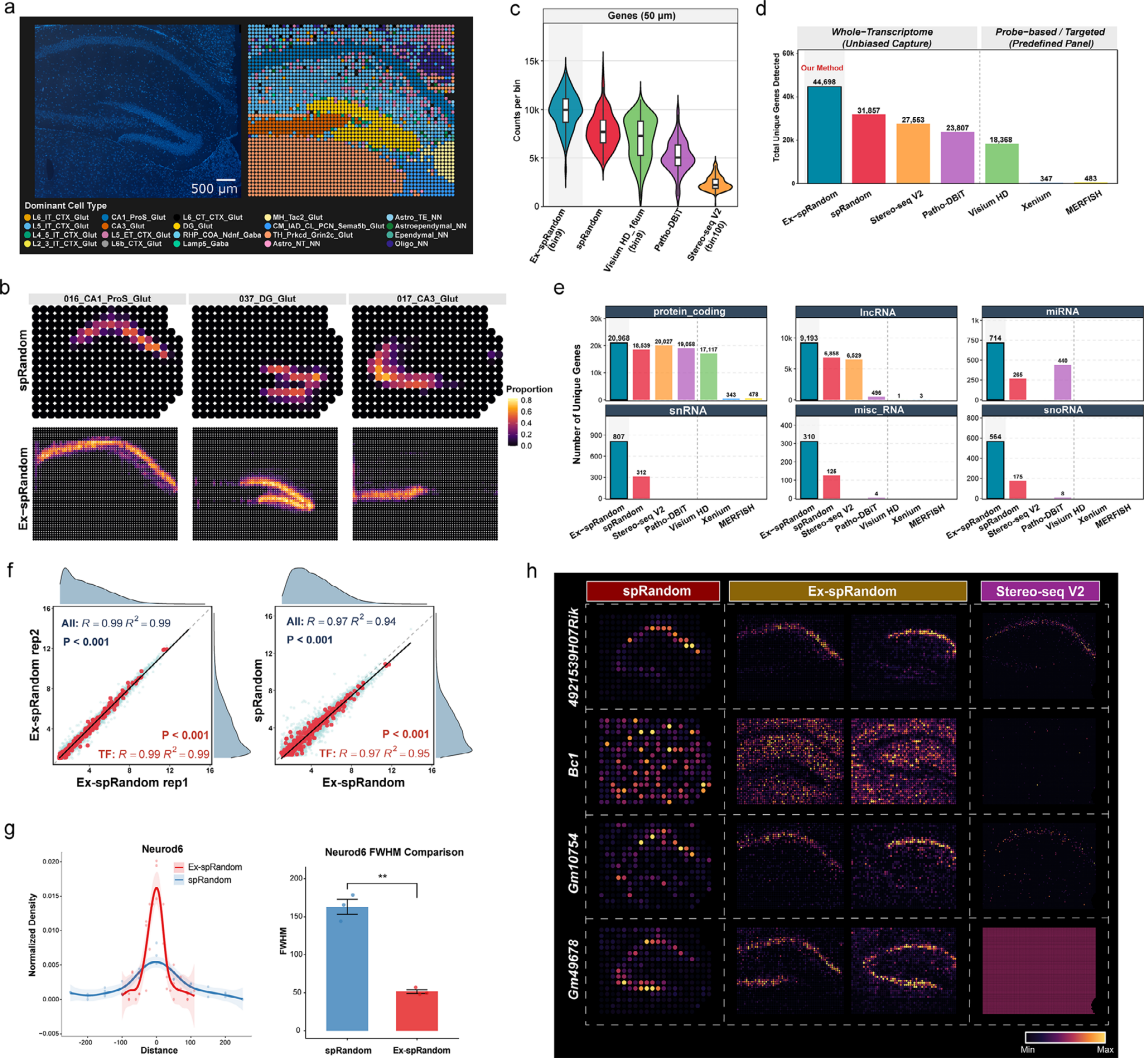
Quantitative benchmarking of Ex‐spRandom for spatial resolution, transcriptomic sensitivity, and signal fidelity. (a) Left: DAPI overview (scale bar, 500 µm). Right: Spatial map of the top 20 dominant cell types in the Ex‐spRandom tissue. (b) Spatial distribution of predicted cell‐type proportions for CA1 ProS, DG, and CA3 glutamatergic neurons in Ex‐spRandom and spRandom tissues. (c) Violin plots of gene capture per 50 µm bin across Ex‐spRandom, spRandom, Visium HD, Patho‐DBiT [10], and Stereo‐seq V2. (d) Bar chart of the total number of unique genes detected across the evaluated spatial transcriptomic datasets and platforms. (e) Number of unique genes detected across six core RNA biotypes for the evaluated platforms. (f) Global transcriptomic correlation of Ex‐spRandom between technical replicates (left) and vs. spRandom (right). Transcription factors (TFs) are highlighted in red; all other filtered genes are in blue. Pearson correlation coefficients (R) and R^2^ values are shown (*p* < 0.001). Marginal histograms indicate gene expression density distributions. (g) Spatial intensity profiling of the neuronal marker *Neurod6*. Left: normalized *Neurod6* expression profiles centered at the peak expression position and scaled to the original tissue distance. Solid lines indicate smoothed fits with 95% confidence intervals. Right: full width at half maximum (FWHM) of the *Neurod6* profiles. Each point represents one selected ROI. h) Cross‐platform spatial mapping of regulatory lncRNAs (*4921539H07Rik*, *Bc1*, *Gm10754*, and *Gm49678*) in spRandom, Ex‐spRandom, and Stereo‐seq V2.

A persistent challenge in spatial transcriptomics is lateral molecular diffusion. To validate the efficacy of our interpenetrating polymer network (IPN) in physically anchoring cDNA, we analyzed the spatial intensity profiles of the neuronal marker *Neurod6*. Ex‐spRandom exhibited a sharply defined intensity gradient across anatomical boundaries, quantitatively supported by a reduced Full Width at Half Maximum (FWHM) compared to conventional unexpanded arrays (Figure 3g and Figure S4a). To further assess this effect, we extended the FWHM analysis to the additional marker genes *Wfs1*, *Grik4*, and *Fibcd1*. Across these markers, Ex‐spRandom showed lower FWHM values than spRandom‐seq after conversion to the original tissue scale (Figure S4). These FWHM measurements provide an empirical comparative assessment of spatial signal sharpness and lateral signal spreading, rather than a direct measurement of molecular resolution. This improved spatial confinement reduced signal overlap between neighboring cell populations; for instance, the Mutually Exclusive Co‐expression Rate (MECR) [30] between distinct populations (e.g., *Prox1+* granule cells and *Gfap+* astrocytes) was reduced (Figure S5). As a result, Ex‐spRandom precisely recapitulates the gold‐standard in situ hybridization (ISH) boundaries [31] (Figure S6). Furthermore, we evaluated the spatial mapping of regulatory non‐coding RNAs, including *4921539H07Rik*, *Bc1*, *Gm10754*, and *Gm49678* (Figure 3h). Ex‐spRandom enabled improved detection of these low‐abundance transcripts and revealed their anatomical enrichment patterns, which were less apparent in the analyzed spRandom and Stereo‐seq V2 datasets. Together, these findings demonstrate that Ex‐spRandom provides improved spatial signal confinement and enhanced capacity for mapping low‐abundance transcripts in FFPE tissues.

## 5 Scalable Iterative Expansion of Ex‐spRandom Enhances Transcriptomic Discovery and Spatial Resolution

The scalable architecture of Ex‐spRandom allows for iterative physical expansion, directly translating into increased transcriptomic information density and enhanced effective spatial resolution. To demonstrate this scalability, we performed iterative expansion on FFPE mouse embryonic eye sections, comparing non‐expanded, first expanded, and second expanded states (Figure 4a). Corresponding DAPI images of the first‐ and second‐expansion samples after expansion are provided in Figure S7, with magnified views showing preservation of the overall ocular morphology and local tissue architecture. Spatial clustering and anatomical annotation revealed that increasing the expansion factor progressively refined ocular architecture. In the non‐expanded sample, although the overall eye structure was discernible, individual cell layers remained spatially compressed (Figure 4a). Upon first and second expansion, we observed improved spatial separation within the resolution of the developing retina, specifically resolving the distinct layers of Retinal Progenitor Cells (RPCs) and Retinal Ganglion Cells (RGCs), alongside the corneal mesenchyme (CM) and corneal epithelium. Notably, while the lens was present in the non‐expanded section, its absence in the first and second expanded samples was attributed to anatomical variations across sectioning planes rather than technical limitations. Despite these sectional differences, the second expanded samples achieved improved structural clarity, transforming previously crowded tissue interfaces into sharply defined cellular landscapes and establishing a foundation for high‐order spatial analysis. Beyond spatial resolution, we evaluated molecular capture performance across the expansion stages. Quantitative analysis revealed a progressive increase in detected genes and unique molecular identifier (UMI) counts that scaled proportionally with the expansion factor (Figure 4b). This enhancement was accompanied by broadened coverage of RNA biotypes, with the second expanded samples recovering a substantially greater variety of both coding and non‐coding RNAs (Figure 4c). Saturation analysis further confirmed that the second expansion yielded more genes than the other groups at equivalent sequencing depths (Figure 4d). Collectively, these results demonstrate that iterative expansion substantially increases transcriptomic data density, providing a comprehensive molecular foundation for in‐depth downstream analyses. Crucially, Ex‐spRandom maintained high transcriptomic fidelity without introducing systematic technical bias. Gene expression consistency was high between different expansion stages (R = 0.986 between first and second) (Figure 4e), and the expanded expression matrices showed strong correlation with the non‐expanded controls (R = 0.960). Furthermore, technical replicates demonstrated exceptional reproducibility (R > 0.96), underscoring the platform's consistent gene detection performance during iterative expansion. We next investigated whether this increased molecular density translated into higher sensitivity for detecting lineage‐specific markers within the developing retina. By comparing the marker genes identified at each stage, we found that iterative expansion significantly broadened the detectable marker repertoire (Figure 4f). Specifically, the second expanded samples uniquely identified 305 retina‐related markers that remained undetected in the non‐expanded or first expanded samples, whereas the non‐expanded dataset showed no stage‐specific retina‐related markers in this comparison (Figure 4f). This leap in sensitivity was exemplified by several key neurodevelopmental genes, such as *Nlgn1* [32], *Dcc* [33], and *Pax6* [34], which exhibited a progressive and significant increase in their transcript capture levels as the expansion factor increased (Figure 4g). To contextualize the biological significance of these second‐unique genes, Gene Ontology (GO) enrichment analysis was performed (Figure 4h). These genes were highly enriched in critical neurobiological processes—including neuron projection guidance, axonogenesis, and synaptic signaling—that are fundamental to eye development but were largely underrepresented in the non‐expanded data [32, 35]. These findings demonstrate that the second expansion improves the detection of developmental gene programs that were less represented in the non‐expanded data. Leveraging the high‐density transcriptomic landscape from the second expansion, we reconstructed the developmental lineage of the embryonic retina with improved continuity. While continuous developmental states were previously obscured by molecular crowding in non‐expanded samples, our expanded dataset unmasked a seamless molecular transition from RPCs to RGCs, consistent with established single‐cell developmental trajectories [32] (Figure 4i and Figure S8a,c). This high‐fidelity mapping elucidated the precise expression dynamics of key neurodevelopmental drivers, such as *Nrxn1* and *Dcx*, which were previously underrepresented (Figure S8b). Furthermore, spatial communication modeling revealed that physical expansion substantially enriches the detectable interactome, yielding an increase in both network complexity and signaling intensity in the second samples (Figure 4j and Figure S9). By resolving core pathways such as NRXN [36] and EPHA [37], we identified spatially organized patterns of critical ligand‐receptor pairs (e.g., *Efna5‐Epha5*) across the retinal architecture. (Figure 4k and Figure S10). Ultimately, these results demonstrate that iterative expansion is an effective strategy for uncovering spatial molecular patterns, thereby deepening our understanding of the regulatory logic of embryonic development.

**FIGURE 4:**
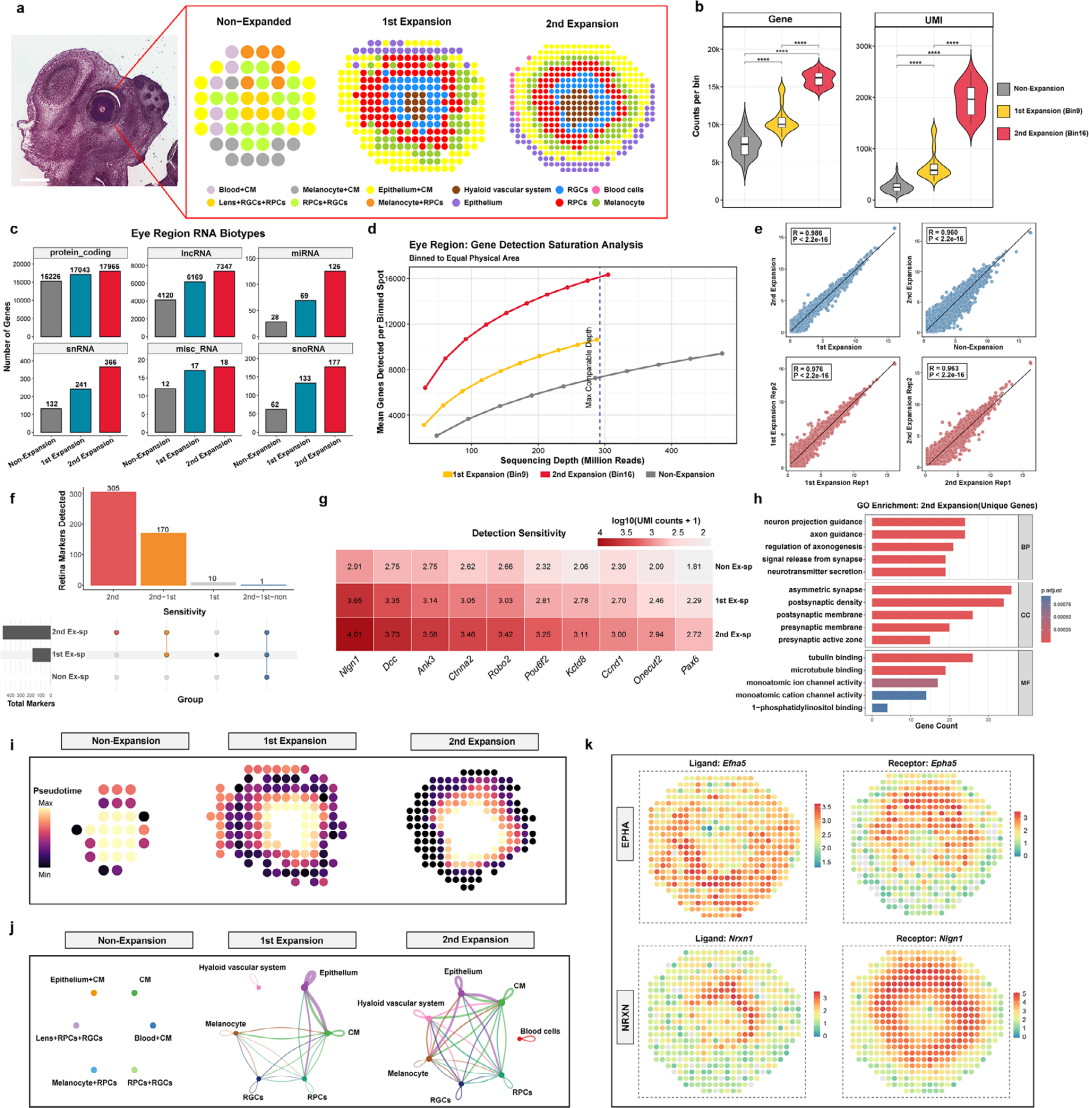
Iterative physical expansion for high‐resolution transcriptomic mapping and developmental modeling. (a) Spatial distribution of annotated cell types in the developing mouse eye. Left: non‐expanded H&E‐stained image (scale bar, 500 µm). Right: corresponding cell‐type maps at non‐expanded, first expansion, and second expansion scales. (b) Quantitative comparison of unique gene and UMI counts per spot across expansion stages. (c) Number of genes detected across major RNA biotypes at equivalent sequencing depths (downsampled to 7813.5 UMIs/spot). (d) Gene detection saturation curves per spatial bin at increasing sequencing depths. The dashed line indicates the unified comparison depth of 292.5 million reads. (e) Scatter plots displaying Pearson correlation (R) values for gene expression profiles (log_2_(CPM + 1)) between technical replicates and expansion stages. (f) UpSet plot illustrating the intersection and unique detection of retina‐related marker genes across expansion groups. (g) Heatmap showing capture levels (log_10_(UMI counts + 1)) of representative neurodevelopmental markers, including Nlgn1 and Pax6, across stages. (h) GO enrichment analysis (Biological Process, BP; Cellular Component, CC; Molecular Function, MF) for genes uniquely detected in the second expansion group. (i) Pseudotime trajectory inference of the retinal lineage from progenitor cells (RPCs) to ganglion cells (RGCs) across expansion scales. (j) Circle plots of inferred global intercellular communication networks showing signaling interaction density for each group. (k) Spatial co‐expression maps for representative ligand‐receptor pairs within the EPHA (*Efna5*‐*Epha5*) and NRXN (*Nrxn1*‐*Nlgn1*) signaling pathways in second expanded tissue.

## 6 Enhanced Spatial Delineation and Host–Microbiome Transcript Detection in FFPE Mouse Colon Section

To evaluate host and microbial transcript detection in FFPE colon, we profiled mouse colon sections using a 2.5‐µm spatial barcode array. For quantitative comparison, we binned the expanded Ex‐spRandom data at level 6, corresponding to a physical bin size of approximately 31 µm in the expanded tissue and an equivalent size of approximately 11 µm in the original tissue scale, and the unexpanded control at level 3 (∼14 µm). These bin sizes were used as comparable spatial analysis units and are distinct from the raw barcode pitch and molecular spatial resolution. Initial mapping of normalized gene detection counts (nGene) revealed a high, uniform molecular capture efficiency across the tissue (Figure 5a). The spatial transcriptomic signals corresponded with major anatomical compartments visible in the DAPI image (Figure 5b). Evaluated at this matched equivalent tissue size, the physical enlargement effectively “unblurred” the transcriptomic map, clearly delineating delicate mucosa, submucosa, and distinct muscle layers (Figure 5b). Consistent with the hippocampal analysis, FWHM measurements of *Clca1*, *Fcgbp*, *Lypd8*, *Muc2*, and *Myh11* showed narrower expression profiles in Ex‐spRandom than in the non‐expanded dataset after conversion to the original tissue scale (Figure S11). These measurements support reduced lateral signal spreading and improved spatial signal confinement in the expanded dataset. (Figure S11). Consequently, this improved spatial delineation supported cell‐type deconvolution via the RCTD algorithm. We successfully resolved 30 distinct host cell populations, mapping specialized lineages like colonocytes and goblet cells strictly to their intricate epithelial architectures (Figure 5c,d and Figure S12). Beyond delineating host cellular architecture, our random‐primed unbiased capture strategy enabled the simultaneous spatial mapping of microbial transcripts within the tissue section. We observed enriched microbial transcript signals primarily within the intestinal lumen and along the epithelial boundary (Figure 5e). Leveraging our established baseline, we evaluated the microbial transcript‐detection sensitivity of Ex‐spRandom. It yielded a median of 23 microbial genes per matched analytical bin (representing an ∼11 µm equivalent tissue size), nearly doubling the detection rate of the unexpanded control (median = 12 genes per ∼14 µm bin) (Wilcoxon test, p < 2.2e‐16; Figure 5f). Furthermore, this enhanced spatial capture translated into an increase in the global microbial transcriptome, pushing the total detected gene pool to over 150,000 unique genes—more than twice the breadth captured without expansion (Figure 5f). This is consistent with physical expansion reducing spatial steric hindrance and improving the accessibility and capture efficiency of fragmented microbial transcripts in highly cross‐linked FFPE tissues. No host or bacterial rRNA depletion was performed. Among microbial reads assigned to annotated rRNA or protein‐coding features, 58.4% were classified as rRNA and 41.6% as protein‐coding transcripts in Ex‐spRandom. Because rRNA annotations are incomplete in some m‐MGnify genomes, the rRNA fraction may be underestimated [38] (Table S3). The relative proportions of predominant microbial species and genera (e.g., *Bacteroides*, *Duncaniella*, and *Muribaculum* [39]) remained broadly comparable between the Ex‐spRandom and non‐expansion datasets, validating the preservation of taxonomic integrity (Figure 5g and Figure S13). The corresponding intestinal‐content sample was also profiled using smRandom‐seq, a microbial single‐cell RNA‐sequencing method for taxonomic identification and transcriptomic profiling [38] (Figure S14a,b). Genus‐level abundance profiles were correlated between Ex‐spRandom and smRandom‐seq (Pearson's R = 0.83, p = 0.0051; Figure S14c). To assess cross‐animal consistency in microbial taxonomic composition, FFPE colon sections from two independent mice raised in the same experimental batch were profiled using spatial arrays with a 15‐µm barcode pitch. The two animals shared similar predominant microbial genera, although differences in their relative abundances were observed (Figure S15). Leveraging this spatial information, we explored localized associations between microbial signals and host transcriptional programs. GO enrichment analysis of host colonocytes spatially co‐localized with specific bacterial taxa (e.g., *Bacteroides* sp002491635) revealed enrichment of transport‐related pathways, including ‘organic acid transport’ and ‘carboxylic acid transport’ [40, 41] (Figure 5h). These results indicate spatial associations but do not by themselves establish direct functional interactions. Collectively, these findings establish Ex‐spRandom as a highly sensitive platform for simultaneously mapping host tissue architecture and microbial transcript signals in FFPE colon tissue.

**FIGURE 5:**
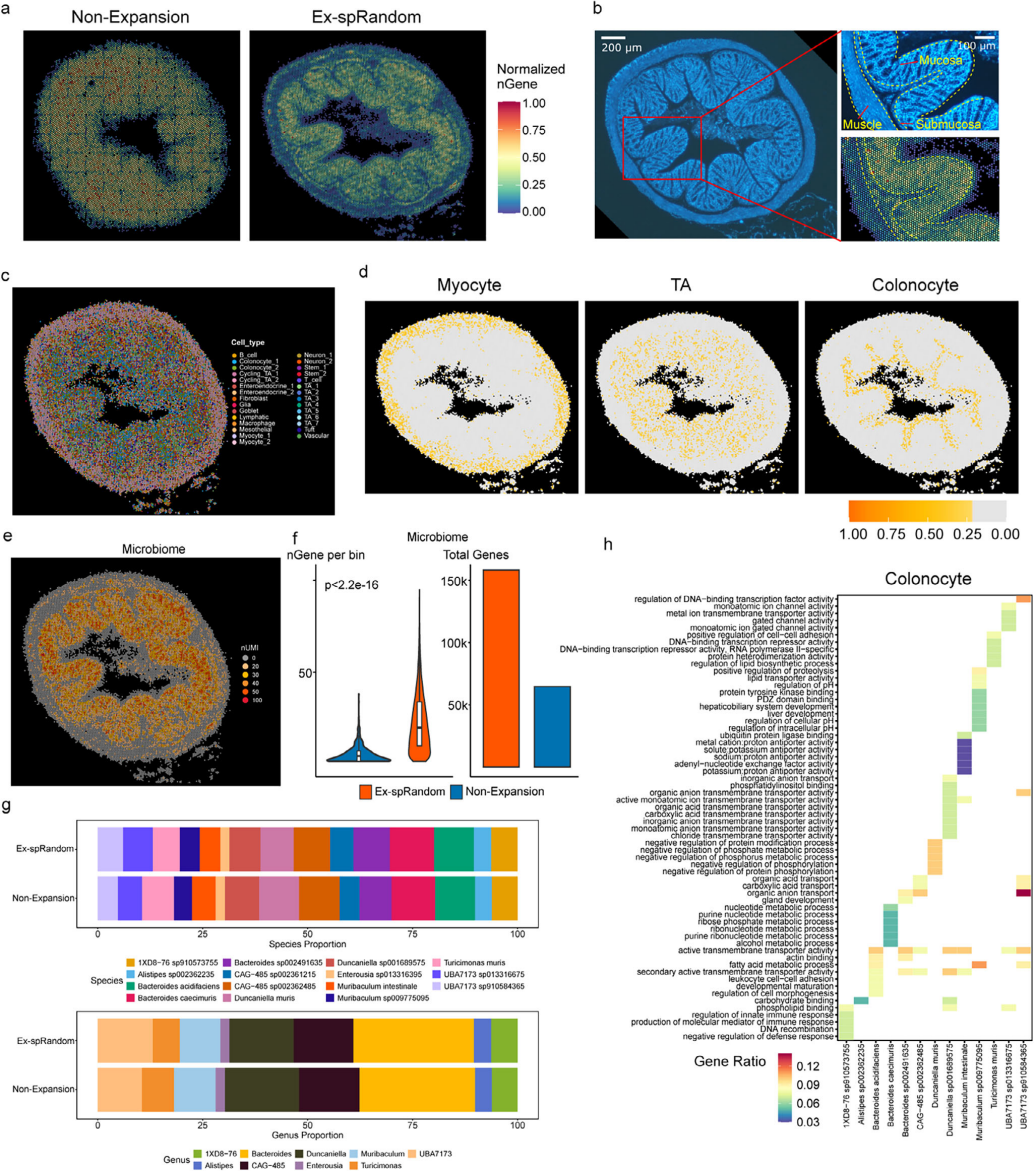
High‐resolution whole‐transcriptome and microbiome profiling of FFPE mouse colon. (a) Normalized gene detection count (nGene) per spatial spot across the colon section. (b) Spatial transcriptomic landscape of the FFPE mouse colon. Left: DAPI overview (scale bar, 200 µm). Top right: Zoom‐in of mucosal architecture (scale bar, 100 µm). Bottom right: High‐resolution transcript map (∼0.9 µm estimated effective barcode pitch). (c) Color‐coded legend of the 30 identified cell types and sub‐clusters. (d) Density scale for cell‐type probability scores (0.00 to 1.00). (e) Spatial distribution of microbiome nUMI counts, showing microbial density across the tissue section. (f) Quantitative comparison of microbial gene detection between Ex‐spRandom and Non‐Expansion. Left panel: Violin plots showing the number of microbial genes captured per spatial bin (nGene per bin) for Non‐Expansion (blue) and Ex‐spRandom (orange), with statistical significance (*p* < 2.2e−16). Right panel: Bar plots showing the total unique microbial genes detected across the tissue for Ex‐spRandom (orange) and Non‐Expansion (blue). (g) Microbial taxonomic composition at the species (top) and genus (bottom) levels, comparing Ex‐spRandom and Non‐Expansion proportions. (h) Dot plot of GO terms enriched in host colonocytes spatially associated with specific microbial species.

## 7 Discussion

In this work, we developed Ex‐spRandom to overcome severe spatial steric hindrance and RNA fragmentation in highly degraded FFPE archival tissues. By rationally synergizing interpenetrating polymer network (IPN) expansion with random‐primed in situ cDNA synthesis, this physical decrowding strategy improves molecular accessibility and spatial signal confinement using accessible spatial barcode arrays. Crucially, Ex‐spRandom enabled a median detection of nearly 10,000 genes per 50‐µm spatial bin under the standardized downstream comparison framework used in this study. This sensitive transcript capture detected over 44,000 host genes and supported the reconstruction of continuous neurodevelopmental trajectories in the embryonic eye. Furthermore, it enabled the simultaneous detection of >150,000 microbial genes alongside host epithelial architecture in the colon.

As summarized in Table S4, previous expansion‐based transcriptomic methods have mainly relied on imaging‐based readouts or poly(A)‐dependent spatial array capture and have largely been demonstrated in non‐FFPE samples. The principal technical advance of Ex‐spRandom lies not in the IPN expansion chemistry itself, but in its integration with an FFPE‐adapted molecular capture and transfer workflow. In Ex‐spRandom, RNA is converted into random‐primed, poly‐dA‐tailed cDNA before hydrogel expansion, and the retained cDNA is subsequently transferred to a spatial barcode array for sequencing. This design reduces dependence on intact poly(A) tails and supports the detection of fragmented, non‐polyadenylated, and microbial transcripts. Hydrogel‐formulation optimization and freeze–thaw‐assisted tissue detachment further facilitate the application of expansion‐based spatial transcriptomics to conventionally processed FFPE sections.

The approximately 0.9‐µm value derived from the array pitch and expansion factor should be interpreted as a geometrically estimated effective barcode pitch rather than a directly measured molecular spatial resolution. Multi‐gene FWHM analyses in the hippocampus and colon showed narrower expression profiles after expansion, consistent with improved spatial signal confinement; however, these measurements provide a comparative assessment of lateral signal spreading rather than an absolute measurement of molecular resolution. In addition, the cross‐platform comparison was based on standardized downstream reanalysis of available datasets rather than fully matched head‐to‐head experiments, and independent biological replication remained limited. For microbiome profiling, the matched smRandom‐seq analysis supported genus‐level taxonomic concordance but did not directly validate the spatial localization of microbial signals. Further evaluation using additional biological replicates, imaging‐based microbial validation, and clinically archived human FFPE tissues will therefore be important. Overall, Ex‐spRandom provides a scalable framework for sensitive spatial profiling of host and microbial transcripts in archival FFPE tissues.

## 8 Methods

### 8.1 Materials and Reagents

10% (v/v) N,N,N',N'‐tetramethylethylenediamine (TEMED), 50% (w/v) acrylamide (AA), 43.8% (w/v) sodium acrylate (SA), and 2% (v/v) N,N‐Dimethylacrylamide (DMAA) were ordered from Adamas. 10% (w/v) ammonium persulfate (APS) was procured from Sigma–Aldrich. 2% (w/v) N,N'‐Methylenebisacrylamide (BIS) was purchased from Innochem. 2% (w/v) N,N'‐Ethylenebisacrylamide (EBIS) was ordered from TCI (Shanghai) Development Co., Ltd. Magnetic beads for purification and single‐stranded DNA library construction kits were supplied by Vazyme. Routine processing reagents, including xylene, graded ethanol solutions (100%, 90%, 70%, 50%, and 30%), standard Hematoxylin and Eosin (H&E) staining reagents, 0.1 m HCl, TE buffer (pH 9.0), 1× PBS, PBST buffer, lysozyme (50 mg/mL), Triton X‐100, 8 M KOH, and 2× SSCT buffer, were obtained from standard commercial sources. Reagents are individually identified in the reagent table, and detailed reaction compositions, reagent volumes, incubation temperatures, and reaction times are provided in the Supplementary Protocol.

### 8.2 Animals and Tissue Preparation

All animal experiments were conducted in accordance with institutional guidelines and approved by the Research Ethics Committee of the First Affiliated Hospital, Zhejiang University School of Medicine (Approval No. 20231446). Female C57BL/6 mice (4 weeks of age; Lingsci Biotechnology, Kunshan, China) were acclimated for one week under standard specific‐pathogen‐free (SPF) conditions, maintained on a 12‐h light/dark cycle with 40%–70% relative humidity at an ambient temperature of 20–26°C. Following the acclimation period, healthy mice were humanely euthanized. Target tissues, including the brain, embryos, and colon, were rapidly harvested under sterile conditions. The collected organs were immediately fixed in 4% paraformaldehyde (PFA) in PBS at 4°C overnight. Following fixation, the tissues were routinely dehydrated and processed into standard formalin‐fixed paraffin‐embedded (FFPE) blocks for subsequent Ex‐spRandom spatial transcriptomic profiling. All in‐house FFPE sections were cut at a thickness of 5 µm. At the time of analysis, the brain and embryonic‐eye FFPE blocks had been stored for approximately 1 year, whereas the colon FFPE blocks had been stored for approximately 1 month.

### 8.3 Tissue‐Free Expansion Test

To systematically evaluate the swelling behavior of different hydrogel formulations, a baseline tissue‐free gel expansion assay was performed. Gel precursor solutions were supplemented with APS and TEMED to final concentrations of 0.1% each, and then dispensed into plastic rings (inner diameter: 8 mm) that were pre‐adhered to glass slides. Following the application of a coverslip, the gels were allowed to polymerize at 37°C for 40 min. Post‐polymerization, the coverslips were removed, the plastic rings were detached, and the pure gels were transferred to 1× PBS for overnight expansion. The expanded gels were collected, laid flat, and imaged using a flatbed scanner. The absolute expansion factor was calculated utilizing the original ring inner diameter as the baseline reference (i.e., expanded gel diameter / 8 mm). Five specific gel formulations (A–E) were evaluated under these controlled conditions, with each condition tested in technical triplicate (*n* = 3).

### 8.4 Histology and Fluorescence Imaging

To acquire spatial morphological references prior to molecular reactions, standard H&E‐stained tissue sections were imaged using a fully automated tissue scanning system (Leica CS2). For nuclear localization, DAPI fluorescence images were acquired on an Olympus CKX53 inverted fluorescence microscope utilizing either a 4× or 10× objective lens. For samples requiring large fields of view, tiled microscopic images were computationally stitched in FIJI (ImageJ) [42] to generate the final high‐resolution composite images.

### 8.5 Contamination Control for Microbial Transcript Detection

For experiments involving microbial transcript detection, tissue processing, enzymatic reactions, cDNA transfer, and library preparation were performed using sterile, nuclease‐free consumables, filtered pipette tips, and nuclease‐free water. Work surfaces and instruments were cleaned before use, and samples were processed separately to minimize experimental contamination.

### 8.6 Tissue Deparaffinization, De‐crosslinking, and Permeabilization

FFPE mouse colon sections were initially baked at 60°C for 2 h to ensure tissue adhesion. The slides were then deparaffinized by immersing them in xylene for 20 min (performed twice), followed by thorough rehydration through a strictly graded ethanol series (100%, 100%, 90%, 70%, 50%, and 30% ethanol) for 3 min each. Following H&E staining for morphology, the sections underwent rigorous de‐crosslinking: tissues were treated with 200 µL of 0.1 m HCl at 42°C for 15 min, rinsed thoroughly with TE buffer (pH 9.0), and subsequently incubated in 200 µL of TE buffer (pH 9.0) at 95°C for 1 h in a thermal cycler. The slides were washed three times with PBST buffer. Tissue permeabilization was then conducted by applying 100 µL of a specialized cell wall digestion reagent (comprising 50 mg/mL lysozyme and 10% Triton X‐100 dissolved in PBS) at 37°C for 30 min. Finally, the slides were washed three times with 2× SSCT buffer to halt the permeabilization.

### 8.7 In Situ Reverse Transcription and Poly‐dA Tailing

Crucially, prior to hydrogel embedding, in situ reverse transcription (RT) was performed directly on the permeabilized tissue slides. A volume of 100 µL of RT reaction mix—consisting of the provided RT buffer, reverse transcriptase (50 U/µL), and 10 mm dNTPs—was applied to the sections. The RT reaction was incubated at 42°C for 90 min to synthesize first‐strand cDNA, followed by three washes with 2× SSCT buffer. To facilitate downstream spatial capture on the transcriptomics array, a poly‐dA tail was added to the synthesized cDNA. This was achieved by applying 100 µL of a tailing reaction mix containing 10× TdT buffer, 10× CoCl_2_ buffer, 100 mM dATP, and Terminal Deoxynucleotidyl Transferase (20,000 U/mL). The tailing reaction was carried out at 37°C for 30 min, and the slides were subsequently washed three times with 2× SSCT buffer.

### 8.8 FFPE Tissue Expansion

For FFPE sections mounted on adhesive slides, a pre‐freezing step was applied immediately before gel incubation: slides were placed at −80°C for 15–30 min, briefly rinsed once with PBS, incubated in PBS at room temperature for 3 min, and then the PBS was removed. (For sections on non‐adhesive slides, this pre‐freezing step was omitted). The tissue sections containing the in situ synthesized cDNA were first incubated with a gel precursor solution lacking APS and TEMED at 4°C for 20 min to ensure thorough infiltration, after which the solution was removed. A 3‐mm‐high metal ring was placed precisely around the tissue, and a coverslip was positioned on top of the ring. A fully activated gel solution containing APS and TEMED was then introduced from the side of the coverslip to completely submerge the tissue. Samples were allowed to equilibrate at 4°C for 20 min and then polymerized at 37°C for 40 min. After carefully removing the coverslip, the gel‐embedded tissue was transferred to 50 mL of PBS and allowed to expand at 4°C. Following physical expansion, excess gel was mechanically trimmed, retaining only the region of interest for spatial capture

### 8.9 Secondary Expansion

Starting from tissue‐containing gels that had already undergone the first expansion step, samples were incubated in stability‐gel monomer solution (monomer2; 4% AA:BIS, 19:1; without APS/TEMED) at 4°C for 20 min. The solution was removed and replaced with monomer2 containing 0.1% APS and 0.1% TEMED, followed by incubation at 4°C for 20 min and polymerization at 37°C for 20 min. Excess peripheral monomer 2 gel was then trimmed away. Samples were subsequently incubated in expansion‐gel monomer solution (monomer1; without APS/TEMED) at 4°C for 20 min. The tissue‐facing side of the gel was then placed tightly against a coverslip; vacuum grease was applied to the four corners of the coverslip, and the assembly was inverted onto a glass slide so that the non‐tissue side also tightly contacted the slide surface. Monomer1 containing 0.1% APS and 0.1% TEMED was then introduced, followed by incubation at 4°C for 20 min and polymerization at 37°C for 40 min. After trimming excess gel, samples were immersed in 1× PBS for expansion until no further obvious size change was observed, and were then used for downstream cDNA transfer.

### 8.10 cDNA Transfer from Expanded Gel to Chip

Following tissue expansion and trimming, the non‐tissue side of the gel was securely mounted against a coverslip to provide structural support. A small amount of vacuum grease was applied around the perimeter of the spatial transcriptomics chip to stabilize the assembly and prevent solution leakage. A cDNA transfer solution was added directly onto the tissue‐facing surface of the hydrogel, which was then carefully brought into physical contact with the spatially barcoded chip. Gentle pressure was continuously applied via the supporting coverslip to ensure flat, uniform contact between the expanded tissue‐gel surface and the capture array. The cDNA molecular transfer reaction was performed at 37°C for 30 min.

### 8.11 Extension and Library Construction

Following cDNA transfer, 200 µL of a DNA polymerization mix (comprising DNA polymerase at 2,000 U/mL, 5× reaction buffer, and 10 mM dNTPs) was applied to the chip and incubated at 37°C for 30 min to generate stable double‐stranded DNA. After washing three times with 2× SSCT buffer, the spatially barcoded cDNA libraries were physically released from the capture array by applying 0.08 m KOH. The released reaction liquid was collected and rigorously purified utilizing magnetic beads. Final sequencing libraries were constructed strictly according to the manufacturer's protocol for single‐stranded DNA library construction kits (Vazyme) and subsequently subjected to next‐generation sequencing.

### 8.12 Preprocessing and Alignment of Sequencing Data

To preprocess both the Ex‐spRandom and standard spRandom sequencing data, spatial barcode (BC) and Unique Molecular Identifier (UMI) sequences were initially extracted from the Read 1 (R1) FASTQ files based on their specific structural lengths (20‐bp BC and 8‐bp UMI). Concurrently, adapter sequences and poly‐A tails were trimmed from Read 2 (R2). The processed R2 reads were then mapped to the mouse reference genome (GRCm38/mm10) using the STARsolo aligner (v2.7.10b) [43], executing the parameter –soloStrand Reverse. The raw gene‐by‐spot expression matrices generated by STARsolo were subsequently utilized for all downstream bioinformatic analyses. Spatial coordinates for each spot barcode were computationally reconstructed according to the predefined microfluidic channel configuration of the capture arrays. Finally, background spots devoid of physical tissue were computationally filtered out by cross‐referencing the spatial spot coordinates with the corresponding morphological H&E or DAPI staining images, ensuring that only tissue‐covered regions were retained.

### 8.13 Spatial Transcriptomics Sequencing Data from Other Technologies

For cross‐platform comparison, publicly available mouse brain datasets collected from spRandom, Stereo‐seq V2, Patho‐DBiT, and 10x Visium HD were utilized. The corresponding publications, public data sources, and available sample‐preparation metadata are summarized in Table S5 and the Data Availability section. Where anatomical information was available, the comparison was focused on the hippocampus or hippocampal brain regions. Raw or preprocessed data matrices from these respective platforms were loaded using the Seurat package (v5.1.0) [44] in R (v4.3.3). Gene nomenclature and expression levels were uniformly extracted for standardized downstream comparison of gene and UMI detection at a final 50‐µm analysis bin. For the Stereo‐seq V2 dataset, spatial data was specifically processed using the Stereopy suite (v1.0.0) [14] to extract relevant gene names and expression matrices. Because several public datasets were available only as processed count matrices, this analysis was considered a standardized downstream reanalysis rather than a fully matched raw‐data benchmark.

### 8.14 Data Processing, Clustering, and Spatial Marker Identification

For both the Ex‐spRandom and standard spRandom datasets, strictly annotated tissue spots were processed using Seurat (v5.1.0). The raw count matrices were normalized and variance‐stabilized utilizing a regularized negative binomial regression model via the SCTransform function [45]. The top 3,000 highly variable features were identified to perform principal component analysis (PCA). To robustly cluster the spatial spots, the first 30 principal components (PCs) were utilized to construct a Shared Nearest Neighbor (SNN) graph (FindNeighbors), followed by unsupervised community detection based on the Louvain algorithm (FindClusters, algorithm = 1). Non‐linear dimensionality reduction for visualization was performed using Uniform Manifold Approximation and Projection (runUMAP). Subsequently, differential expression (DE) analysis was executed on the standard RNA assay to accurately evaluate true fold changes. The raw counts were log‐normalized (NormalizeData) and centered (ScaleData). Differentially expressed genes (DEGs) were computed using the Wilcoxon rank‐sum test (FindAllMarkers). Spatial markers were stringently filtered requiring: positive enrichment (only.pos = TRUE), detectable expression in ≥10% of spots (min.pct = 0.1), and a log‐fold change >0.25. Only DEGs demonstrating a false discovery rate (FDR)‐adjusted *p* value < 0.05 (Benjamini‐Hochberg) were retained.

### 8.15 Spatial Deconvolution and Spot Purity Assessment

Cell‐type deconvolution was performed using RCTD via the spacexr package (v2.2.1) [29] to quantitatively estimate cell‐type proportions within each spatial spot. The dominant cell type per spot was assigned based on the highest predicted computational weight. To compare spatial localization fidelity across conditions, normalized weight matrices for target hippocampal cell types (CA1, CA3, and DG) were extracted, and the non‐expanded control coordinates were rotated 180 degrees to align anatomically with the expanded samples. Spot purity was defined as the maximum proportion of a single cell type within a given spot after weight normalization. This purity analysis was specifically restricted to core hippocampal neuronal layers (CA1, CA2, CA3, DG, and SUB). Statistical shifts in spot purity distributions between expanded and non‐expanded groups were visualized using smoothed density plots and evaluated using a Wilcoxon rank‐sum test.

### 8.16 Transcriptomic Benchmarking (Detection Density and Breadth)

Pseudo‐bulk expression profiles were generated by aggregating raw counts across all spatial spots. Technical reproducibility and transcriptomic concordance were assessed by comparing Ex‐spRandom profiles between section‐level technical replicates and with standard spRandom and an external MERFISH dataset. Sequencing data were normalized as log2(CPM+1), while MERFISH data utilized quantile normalization (mitochondrial/ribosomal genes were excluded). Pearson correlation coefficients (R) and R^2^ values were calculated to evaluate transcriptomic concordance. Transcript capture density was calculated as UMIs and unique genes per 50 × 50 µm^2^ spatial bin. Raw counts were aggregated into uniform 50 µm bins across platforms. For Ex‐spRandom, coordinates were adjusted using an effective pitch of 50/3 µm (linear expansion factor of 3) to reflect pre‐expansion tissue area. Density distributions were visualized with violin plots overlaid with boxplots. Spatial bins were treated as measurement units for technical comparison rather than as independent biological replicates.

### 8.17 Biotype Classification and Composition Analysis

Detected gene symbols were strictly mapped to a reference mouse gene annotation database to determine transcript biotypes (e.g., protein‐coding, lncRNA, miRNA, snRNA). Unique gene symbols from Ex‐spRandom and standard spRandom were intersected and visualized using an Euler diagram. The biotype composition of total gene pools and the specific subset uniquely detected by Ex‐spRandom was calculated as relative percentages, isolating pseudogenes and TEC transcripts to highlight shifts in detection profiles.

### 8.18 Gene Detection Saturation Analysis

To systematically evaluate depth‐dependent gene discovery rates, saturation analysis was performed using a binomial downsampling approach. For each binned unit, UMI counts in the gene‐by‐bin count matrix were randomly sampled at diverse fractions (10% to 100%) of observed count depth. The mean number of genes detected was projected against total sequencing reads plotted against the corresponding sequencing depth, calculated using the quality‐filtered reads used for alignment (“reads to align”) for each library. per binned unit was projected against total sequencing reads plotted against the corresponding sequencing depth, calculated using the quality‐filtered reads used for alignment (“reads to align”) for each library. A unified comparison threshold of 292.5 million reads was established for equal‐depth comparison across groups. This threshold corresponded to the lowest reads‐to‐align depth among the libraries included in the saturation analysis, and libraries with higher sequencing depth were compared up to this common depth.

### 8.19 Quantification of Molecular Diffusion and Spatial Crosstalk

To comparatively assess spatial signal confinement, one‐dimensional expression profiles were extracted across anatomically defined boundaries. Hippocampal markers included *Neurod6*, *Wfs1*, *Grik4*, and *Fibcd1*. For each selected rectangular region, expression values were aggregated along the axis parallel to the cell layer and represented as a function of perpendicular distance from the expression boundary. The resulting profiles were normalized and smoothed using LOESS regression. FWHM was calculated as the distance between the two positions at which the smoothed profile reached half of its maximum value. For colon markers, including *Clca1*, *Fcgbp*, *Lypd8*, *Muc2*, and *Myh11*, a reference curve was defined along the corresponding anatomical structure. Spatial measurement units within a predefined perpendicular distance corresponding to 300 µm at the original tissue scale were projected onto the curve, and mean expression was calculated as a function of perpendicular distance. The resulting profiles were normalized and smoothed, and FWHM was determined from the half‐maximum crossing positions. For expanded samples, distances and FWHM values were divided by the measured linear expansion factor of the corresponding sample to convert the measurements to the original tissue scale. FWHM was used as a comparative measure of spatial signal confinement and lateral signal spreading rather than as a direct measurement of molecular diffusion or absolute molecular resolution. Spatial signal contamination was quantified using the Mutually Exclusive Co‐expression Rate (MECR) [30] based on non‐overlapping markers (Prox1 and Gfap). Expression was binarized (log1p counts >1 threshold). The MECR was calculated as the percentage of spots co‐expressing both markers relative to the total number of spots expressing either gene, visualized via 2D scatter plots overlaid with density contours.

### 8.20 Spatial Visualization and ISH Benchmarking

Spatial expression patterns were visualized by projecting log1p‐normalized UMI counts (capped at the 99th percentile) onto aligned tissue coordinates. To facilitate accurate cross‐platform architectural comparisons, coordinate matrices for spRandom, Ex‐spRandom, and Stereo‐seq V2 were computationally transposed or rotated to maintain strict anatomical orientation. Corresponding in situ hybridization (ISH) reference images were sourced from the Allen Brain Atlas to visually benchmark spatial boundary fidelity.

### 8.21 Retina‐Specific Marker Identification and Quantitative Analysis

Retina‐specific marker genes were identified utilizing Seurat by comparing retina‐related clusters (RPCs, RGCs, and mixed types) against others (logFC >0.25, adjusted P <0.05, expressed in >25% of spots). Intersections of these gene sets across second expansion, first expansion, and non‐expansion datasets were visualized using ComplexUpset. For quantitative sensitivity assessment, raw UMI counts of 10 representative retinal genes were aggregated, transformed as log10(UMI+1), and visualized using pheatmap. Furthermore, Gene Ontology (GO) functional enrichment analysis on uniquely detected genes was performed using clusterProfiler [46], converting symbols to Entrez IDs via org.Mm.eg.db.

### 8.22 Development Trajectory and Spatial Cell‐Cell Communication

Retinal maturation trajectories were reconstructed using Monocle3 [47] by extracting RPC/RGC subsets and converting them into cell_data_set objects. Following UMAP reduction, the developmental root was assigned to the spot with maximum Sox2 expression, and pseudotime was calculated via geodesic distance along the learned principal graph. Trajectory‐dependent genes were identified via Moran's I test (q < 0.05) and fitted using LOESS regression. Concurrently, cell‐cell communication was modeled using CellChat (v2) [48] with a truncated mean strategy (trim = 0.1), considering spatial contact ranges adjusted by expansion scale. Key signaling pathways (SEMA3, EPHA, LAMININ) were visualized using spatial network diagrams to validate molecular co‐localization.

### 8.23 High‐resolution Spatial Host‐microbiome Transcriptomic Analysis

Raw spatial host‐microbiome sequencing files were robustly analyzed using BSTMatrix (v2.4.f.4). The raw data were initially aligned to the host genome (Mouse GRCm39). Unmapped reads were extracted and subsequently aligned to a customized merged microbial genome. To identify the microbial taxa present in the corresponding intestinal‐content sample, smRandom‐seq data were analyzed using the MIC‐Anno module of MICtools against the MGnify Genomes Mouse Gut catalogue v1.0 (m‐MGnify) [49]. Microbial cells with more than 500 UMIs were retained, and barcodes were further filtered when the dominant species accounted for more than 10% of the classified reads. Based on the taxa identified by smRandom‐seq, the corresponding representative genomes and annotations were selected from m‐MGnify and merged to construct the customized microbial reference. A STAR index was generated from the merged genome FASTA and GTF files using STAR (v2.7.10a) with –sjdbOverhang 122. Spot binning was performed with BSTMatrix (level‐6 for Ex‐spRandom; level‐3 for non‐expansion). Because closely related microbial genomes may produce ambiguous species‐level assignments, microbial composition comparisons were primarily performed at the genus level. Unique‐ and multi‐mapping fractions were calculated among reads aligned to the customized microbial reference. No host or bacterial rRNA depletion was performed. rRNA and protein‐coding fractions were calculated among microbial reads assigned to either annotated rRNA or protein‐coding features. For cross‐platform taxonomic comparison, genus‐level relative abundances detected by Ex‐spRandom were compared with those obtained from the matched smRandom‐seq dataset generated from the corresponding intestinal‐content sample using Pearson correlation analysis. Spatial matrices were processed with Seurat for clustering, and cell‐type deconvolution was performed using RCTD (doublet mode). To deeply investigate functional differences within TA, colonocyte, and myocyte cells driven by distinct bacterial taxa, tissue sections were dichotomized into “high” and “low” microbial‐signal regions based on min‐max normalized microbial UMI counts (Threshold = 0.25). Target host cells spatially co‐localized within species‐specific “high” regions were grouped, and localized Differentially Expressed Genes (DEGs) were identified via Seurat's FindAllMarkers. These DEGs were finally utilized to perform Gene Ontology enrichment tests via the enrichGO function in the clusterProfiler R package.

### 8.24 Statistical Analysis and Definition of Replicates

Spatial spots or binned units from the same tissue section were treated as spatial measurement units rather than independent biological replicates. Adjacent sections obtained from the same animal and FFPE block and processed on separate spatial arrays were considered section‐level technical replicates, whereas samples obtained from different animals were considered biological replicates. Statistical comparisons based on spots or bins were used to evaluate within‐section technical differences and were not interpreted as animal‐level biological inference. The numbers of animals, tissue sections, arrays, and replicate types for each dataset are summarized in Table S6. Statistical tests used for individual analyses are specified in the corresponding Methods sections and figure legends, and *p* < 0.05 was considered statistically significant.

## Author Contributions

**Yuyi Zhu**: methodology, validation. **Ziye Xu**: conceptualization, funding acquisition. **Bin Zhu**: methodology. **Shunji Zhang**: conceptualization, methodology, data curation, writing – original draft, visualization, validation. **Yongcheng Wang**: writing – review and editing, resources, supervision, conceptualization, investigation, project administration, formal analysis. **Mengyuan Wang**: methodology, visualization, writing – original draft, validation, investigation. **Yuan Liao**: conceptualization, writing – review and editing, supervision. **Jiaye Chen**: software, data curation, validation, visualization, investigation.

## Funding

The project was supported by the Noncommunicable Chronic Diseases‐National Science and Technology Major Project (2026ZD0556100, Y.W.), the Hangzhou Leading Innovation and Entrepreneurship Team Project (TD2024015), and the Key R&D Program of Zhejiang (2024SSYS0022, Y.W.).

## Conflicts of Interest

Yongcheng Wang is a co‐founder of M20 Genomics.

## Supporting information




**Supporting File 1**: advs77219‐sup‐0001‐SuppMat.docx.

**Supporting File 2**: advs77219‐sup‐0002‐SuppMat.pdf.

## Data Availability

The spatial transcriptomics data generated in this study are available in the NCBI Gene Expression Omnibus (GEO) database under the accession code GSE324753 (https://www.ncbi.nlm.nih.gov/geo/query/acc.cgi?acc = GSE324753). Publicly available spatial transcriptomics datasets used for benchmarking were obtained from official data portals or public repositories. Specifically, the Visium HD adult mouse brain coronal section dataset is available from the 10x Genomics official website (https://www.10xgenomics.com/datasets/visium‐hd‐cytassist‐gene‐expression‐libraries‐of‐mouse‐brain‐he‐v4). The Stereo‐seq V2 mouse brain dataset can be accessed via the STOmics resource portal (https://www.stomics.tech/col1187). The MERFISH mouse brain receptor map dataset was obtained from the Vizgen official data portal (https://info.vizgen.com/mouse‐brain‐map). The wild‐type mouse brain Xenium in situ dataset is available via the 10x Genomics dataset repository (https://www.10xgenomics.com/datasets/xenium‐in‐situ‐analysis‐of‐alzheimers‐disease‐mouse‐model‐brain‐coronal‐sections‐from‐one‐hemisphere‐over‐a‐time‐course‐1‐standard). Furthermore, the spatial transcriptomics datasets for spRandom and Patho‐DBiT were retrieved from GEO under accession numbers GSE247881 and GSE274641, respectively.
