# Supplementary material for: Highly Sensitive Spatial Host‐Microbiome Transcriptomics in FFPE Tissues via Iterative Hydrogel Expansion
Source: Adv Sci (Weinh). 2026 Aug 17:e77219. Online ahead of print. doi: 10.1002/advs.77219 (PMC13479583; doi:10.1002/advs.77219)
Supplement: Supplementary file 1 [file ADVS-9999-e77219-s002.pdf]

Supporting Information

Highly sensitive spatial host-microbiome transcriptomics in FFPE tissues via iterative hydrogel expansion

Shunji Zhang, Mengyuan Wang, Jiaye Chen, Yuyi Zhu, Bin Zhu, Ziye Xu, Yuan Liao, Yongcheng Wang**

Table S1. Formulation of gel recipes

Recipe	SA (% w/v)	AA (% w/v)	BIS (% w/v)	EBIS (% w/v)	DMAA (% v/v)	PBS	Total (μL)
**A**	10.30	14.20	0.06	—	—	1×	1000
**B**	10.30	14.20	0.02	—	—	1×	1000
**C**	10.30	14.20	0.006	—	—	1×	1000
**D**	34.00	10.00	—	0.005	—	1×	1000
**E**	33.20	10.00	0.005	—	4.00	—	1000

Notes:

1. Stock concentrations: SA 43.8% (w/v), AA 50% (w/v), BIS 2% (w/v), EBIS 2% (w/v), DMAA 98% (v/v), PBS 10×.
2. Add water to reach the final volume.

Table S2. Comparison of capture performance between Ex-spRandom and other spatial transcriptomics technologies in FFPE mouse hippocampus samples

	Ex-spRandom (bin9)	spRandom[1]	Patho-DBiT[2]	Stereo-seq V2[3] (bin100)	Visium HD_16um (bin9)
**Median gene (50 µm)**	10007.0	7690	5056.5	2232.5	4463.0
**Median UMI (50 µm)**	111002.0	76853	15914.5	8130.5	10139.0
**Total gene**	44,698	31,857	23,807	27,553	18,368
**Reference / data source**	This study	Liao et al., bioRxiv 2025, 2025.02.19.638981	Bai et al., Cell 2024, 187, 6760	STOmics resource portal	10x Genomics Visium HD adult mouse brain coronal section dataset

Table S3. Microbial mapping and transcript-composition statistics for the colon datasets.

Sample	Analysis bin	uniquely mapping ratio %	multi mapping ratio %	rRNA ratio %	Protein coding ratio %
Ex-spRandom	bin6 (31µm)	49.87	50.13	58.4	41.6
Non-Expansion	bin3 (14µm)	43.40	56.60	52	48

Table S4. Comparison of Ex-spRandom with representative expansion-based transcriptomic methods.

Method	Method category	Demonstrated sample type	Molecule retained during expansion	Main hydrogel components	Proteinase K digestion	Transcript detection strategy
ExSeq[4]	Imaging-based in situ sequencing	Fixed cultured cells and fixed tissue sections	LabelX-anchored native RNA	SA/AA/BIS	Yes	Random primers; predefined padlock probes also supported
Expansion-MERFISH[5]	Imaging-based targeted transcript detection	Fixed cultured cells	Poly(dT)-LNA-anchored poly(A) RNA	SA/AA/BIS	Yes	Predefined gene-specific probes
EASI-FISH[6]	Imaging-based targeted transcript detection	Fixed thick mouse brain sections	MelphaX-anchored native RNA	SA/AA/BIS	Yes	Predefined gene-specific probes
Ex-ST[7]	Sequencing-based spatial transcriptomics	Fresh-frozen mouse brain cryosections	Poly(dT)-LNA-anchored poly(A) RNA	SA/AA/BIS	Yes	Poly(dT)-based capture
Ex-spRandom	Sequencing-based spatial transcriptomics	FFPE tissue sections	In situ synthesized poly-dA-tailed cDNA	SA/AA/BIS/DMAA; optional secondary hydrogel re-embedding	No	Random-primer-based capture

Table S5. Sample preparation metadata for datasets used in cross-platform benchmarking

Platform	Tissue type	Species	FFPE block age	Fixation condition	Fixation duration	Section thickness
Ex-spRandom	Hippocampus	Mus musculus	1 year	4% Paraformaldehyde	24 hr	5 μm
	Embryonic eye	Mus musculus	1 year	4% Paraformaldehyde	24 hr	5 μm
	Colon	Mus musculus	1 month	4% Paraformaldehyde	24 hr	5 μm
spRandom[1]	Hippocampus	Mus musculus	not reported	4% Paraformaldehyde	24 hr	5 μm
Visium HD	Hippocampus	Mus musculus	not reported	not reported	not reported	5 μm
Stereo-seq V2[3]	Hippocampus	Mus musculus	not reported	Neutral Buffered formalin	not reported	5 μm
Patho-DBiT[2]	Hippocampus	Mus musculus	not reported	10% Neutral Buffered formalin	not reported	7-10 μm

Table S6. Sample and replicate information for Ex-spRandom datasets

Figure	Sample name	Tissue type	Animal	Section	Experimental condition
Fig. 3f	Hippo-1st Ex-rep1	Hippocampus	Mouse 1	Adjacent sections	1st expansion
Fig. 3f	Hippo-1st Ex-rep2				1st expansion
Fig. 4	Eye-Ctrl	Embryonic eye	Mouse 2	Adjacent sections	Non-expansion
Fig. 4	Eye-1st Ex-rep1				1st expansion
Fig. 4	Eye-1st Ex-rep2				1st expansion
Fig. 4	Eye-2nd Ex-rep1				2nd expansion
Fig. 4	Eye-2nd Ex-rep2				2nd expansion
Fig. 5	Colon1-Ctrl	Colon	Mouse 3	Adjacent sections	Non-expansion
Fig. 5	Colon1-1st Ex-rep1				1st expansion
Fig.S15	Colon1-1st Ex-rep2				1st expansion
Fig.S15	Colon2-1st Ex		Mouse 4	Individual section	1st expansion

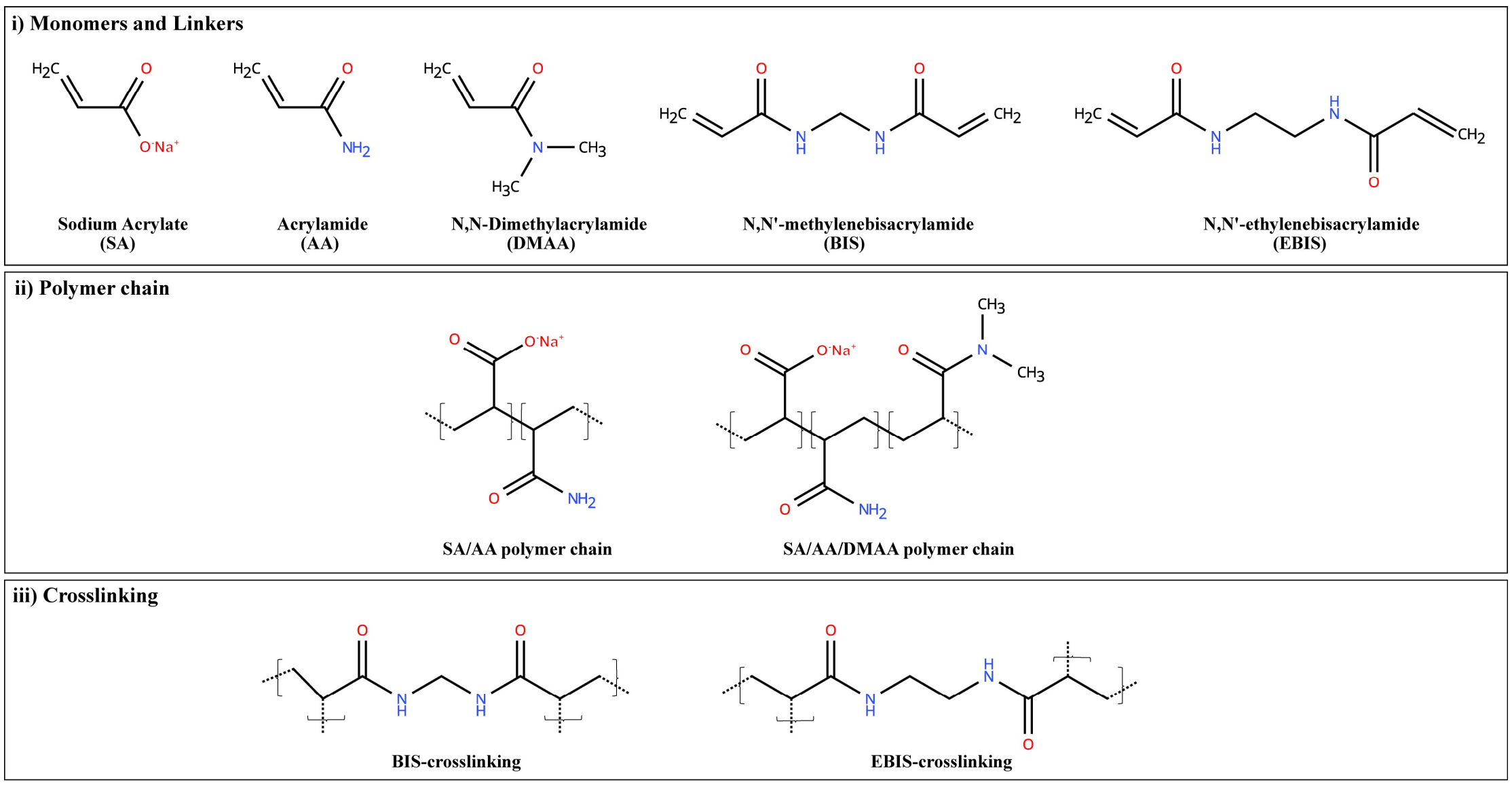

Figure S1 Components and structures of the expandable hydrogels. (i) Molecular structures of monomers (SA, AA, DMAA) and crosslinkers (BIS, EBIS) used in this study. (ii) Representative chemical structures of the uncrosslinked SA/AA and SA/AA/DMAA polymer chains. (iii) Schematic illustration of the crosslinked network structures (BIS-crosslinking and EBIS-crosslinking) forming the hydrogel scaffold.

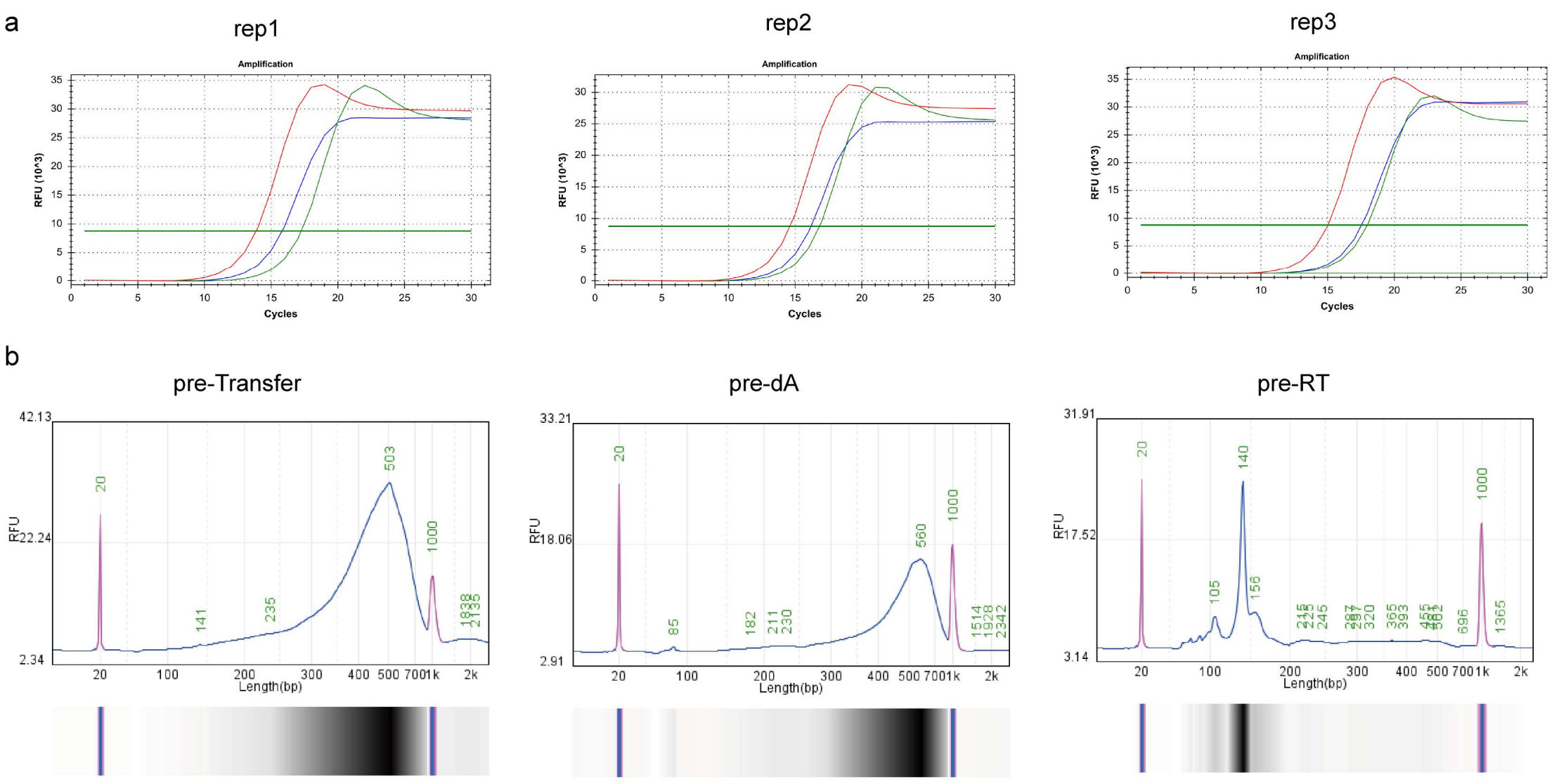

Figure S2. qPCR and qsep analyses under pre-RT, pre-dA, and pre-Transfer conditions. a) qPCR fluorescence curves from three groups, each including three conditions (Blue, pre-RT; green, pre-dA; red, pre-Transfer). b) qsep profiles for the corresponding three conditions.

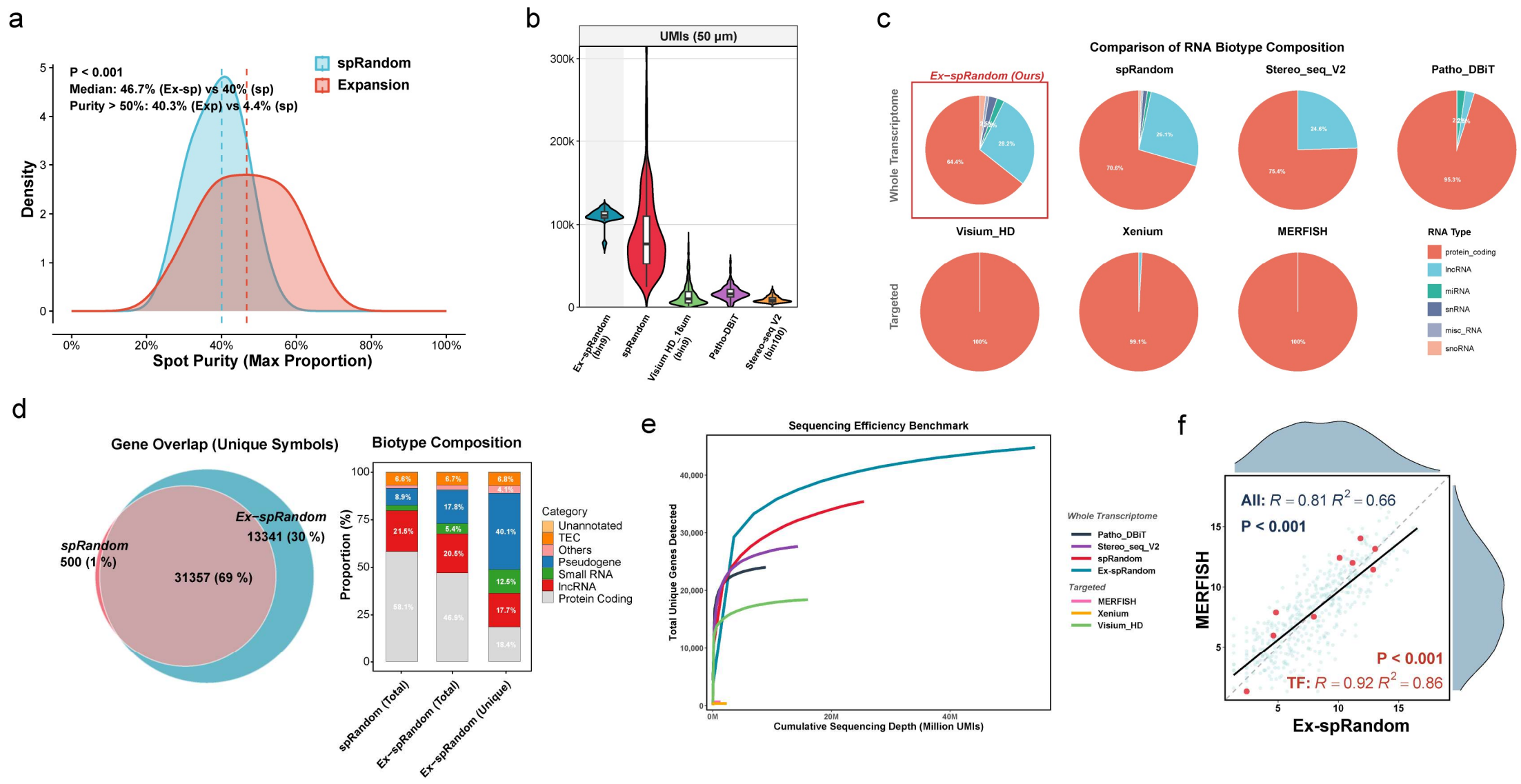

Figure S3. Enhanced spatial purity and transcriptomic coverage of Ex-spRandom. a) Density plot of spot purity (defined as the maximum cell-type proportion per spot) in core hippocampal neuronal layers for Ex-spRandom (red) and spRandom (blue). Median purity and the fraction of high-purity spots (>50%) are annotated ($P < 0.001$, Wilcoxon rank-sum test). b) Violin plots of UMI capture densities per 50 μm across Ex-spRandom, spRandom, Visium HD, Patho-DBiT, and Stereo-seq V2. c) Stacked bar charts detailing the relative proportions of captured RNA biotypes (e.g., protein-coding, lncRNA, miRNA) across the evaluated technologies. d) *Left:* Euler diagram showing the intersection of detected gene symbols between spRandom and Ex-spRandom. *Right:* Stacked bar charts of the relative biotype composition for total detected genes and genes uniquely detected by Ex-spRandom. e) Sequencing saturation curves displaying the total unique genes detected as a function of cumulative sequencing depth (Million UMIs) for Ex-spRandom, conventional whole-transcriptome, and targeted spatial platforms. f) Global transcriptomic correlation of Ex-spRandom versus a reference MERFISH dataset. Transcription factors (TFs) are highlighted in red. Pearson correlation coefficients (R) and R^2 values are shown ($P < 0.001$). Marginal histograms indicate gene expression density distributions.

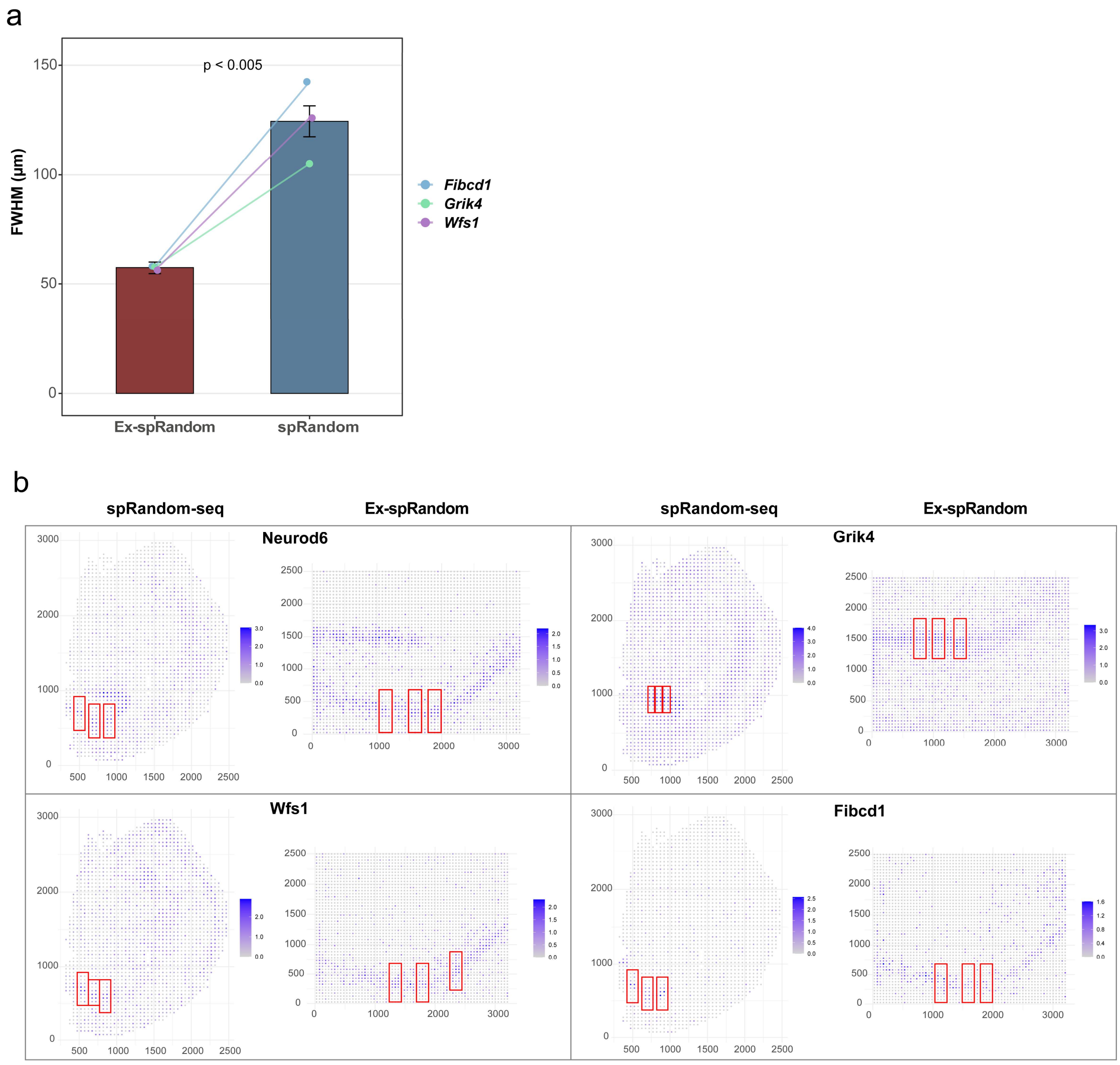

Figure S4. FWHM analysis of spatial expression profiles in the mouse hippocampus. a, Comparison of FWHM values for *Fibcd1*, *Grik4*, and *Wfs1* between Ex-spRandom and spRandom-seq datasets generated using a 50-µm spatial barcode array. Colored points indicate individual genes, with paired measurements connected by lines. b, Spatial expression patterns of *Neurod6*, *Grik4*, *Wfs1*, and *Fibcd1*. Red boxes indicate regions used for FWHM analysis. All measurements were converted to the original tissue scale.

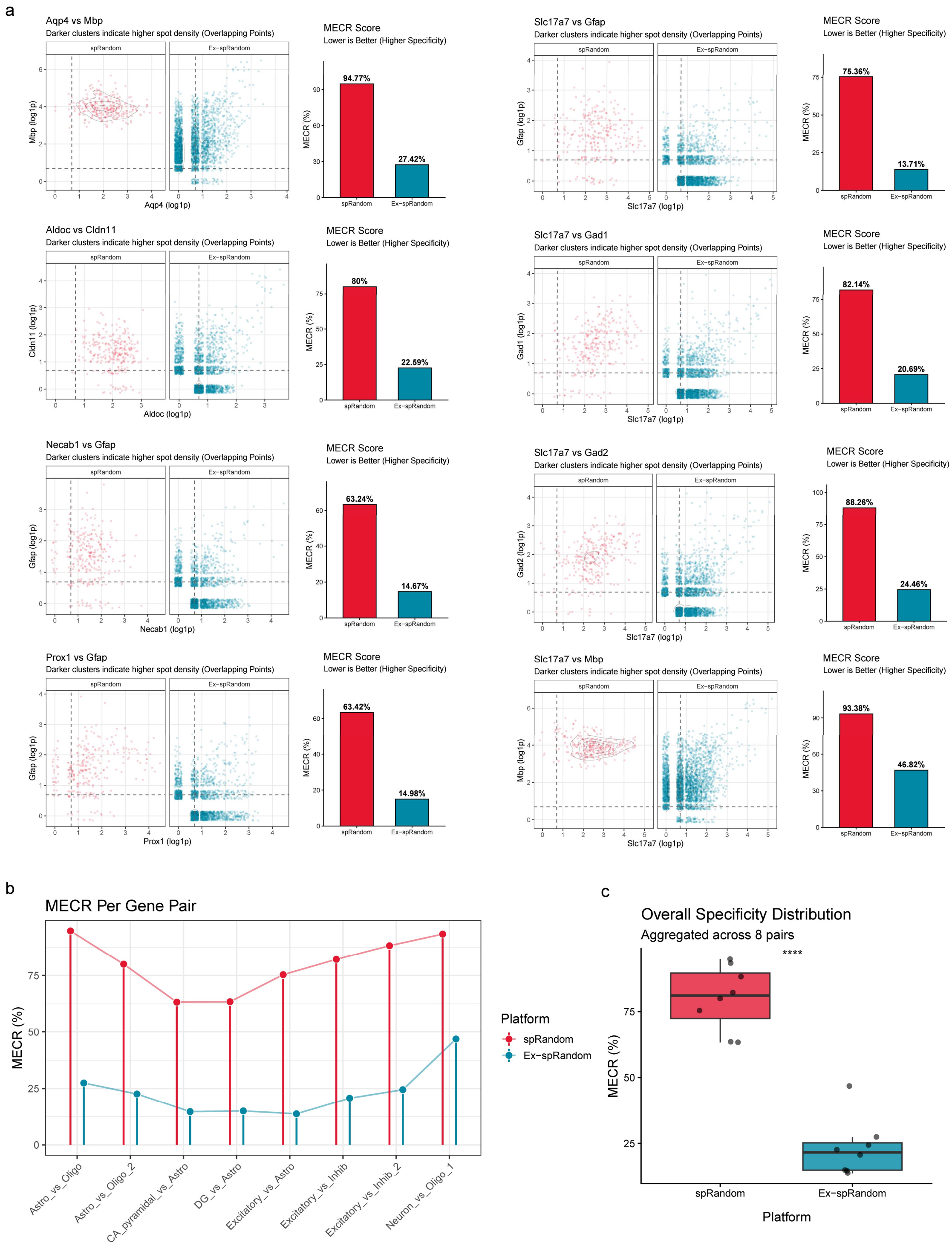

Figure S5. Spatial signal cross-contamination analysis. a) Single-spot co-expression scatter plots for representative mutually exclusive cell-type marker pairs (e.g., *Aqp4* vs. *Mbp*, *Aldoc* vs. *Cldn11*, *Prox1* vs. *Gfap*) in unexpanded spRandom and Ex-spRandom. b) Quantitative comparison of the Mutually Exclusive Co-expression Rate (MECR) across the evaluated gene pairs. c) Boxplot showing the overall MECR distribution aggregated across all 8 gene pairs for spRandom and Ex-spRandom (**** $P < 0.0001$).

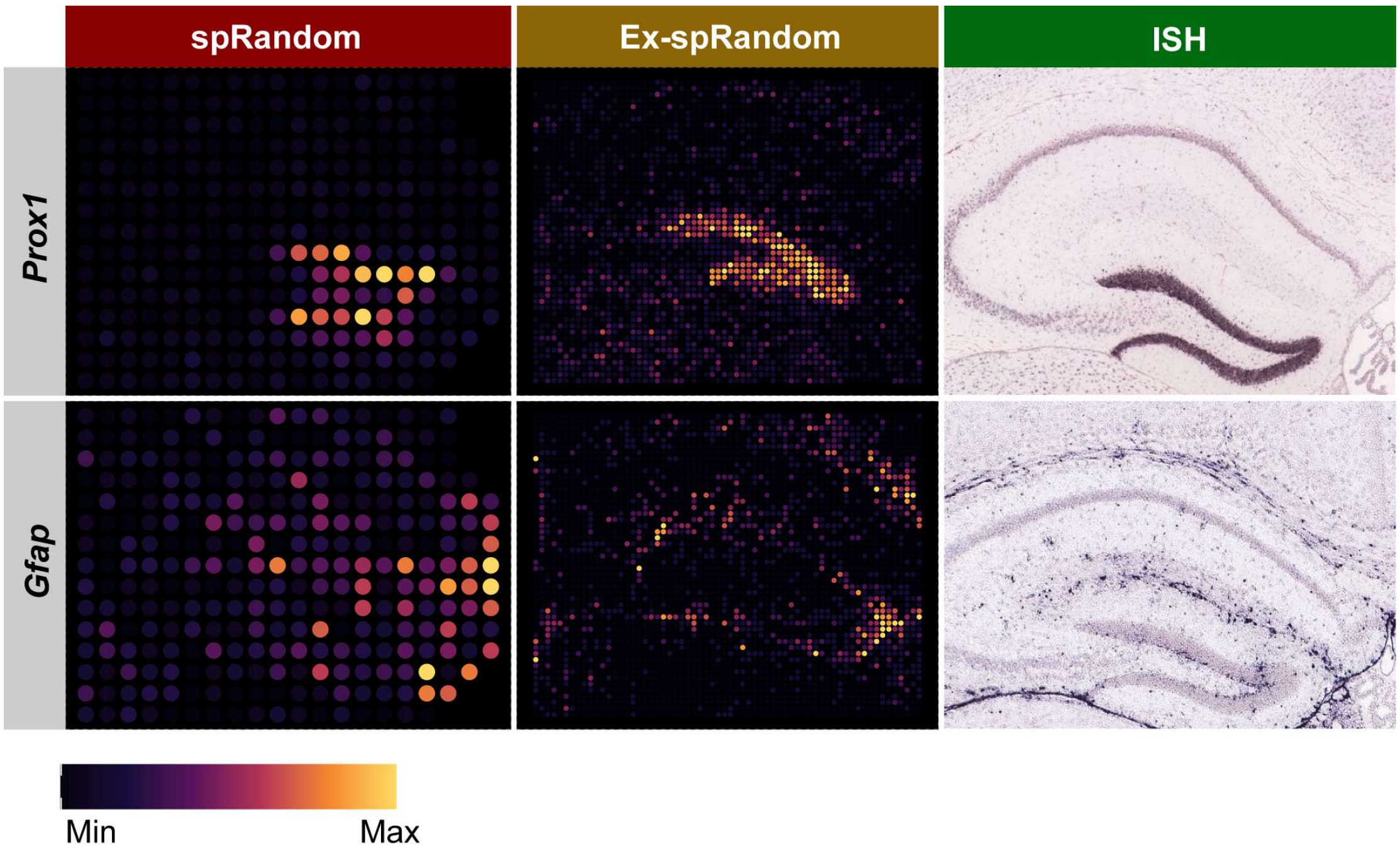

Figure S6. Validation of spatial boundaries. Spatial expression maps of *Prox1* and *Gfap* in the hippocampal dentate gyrus for spRandom and Ex-spRandom, directly compared alongside Allen Brain Atlas in situ hybridization (ISH) reference images.

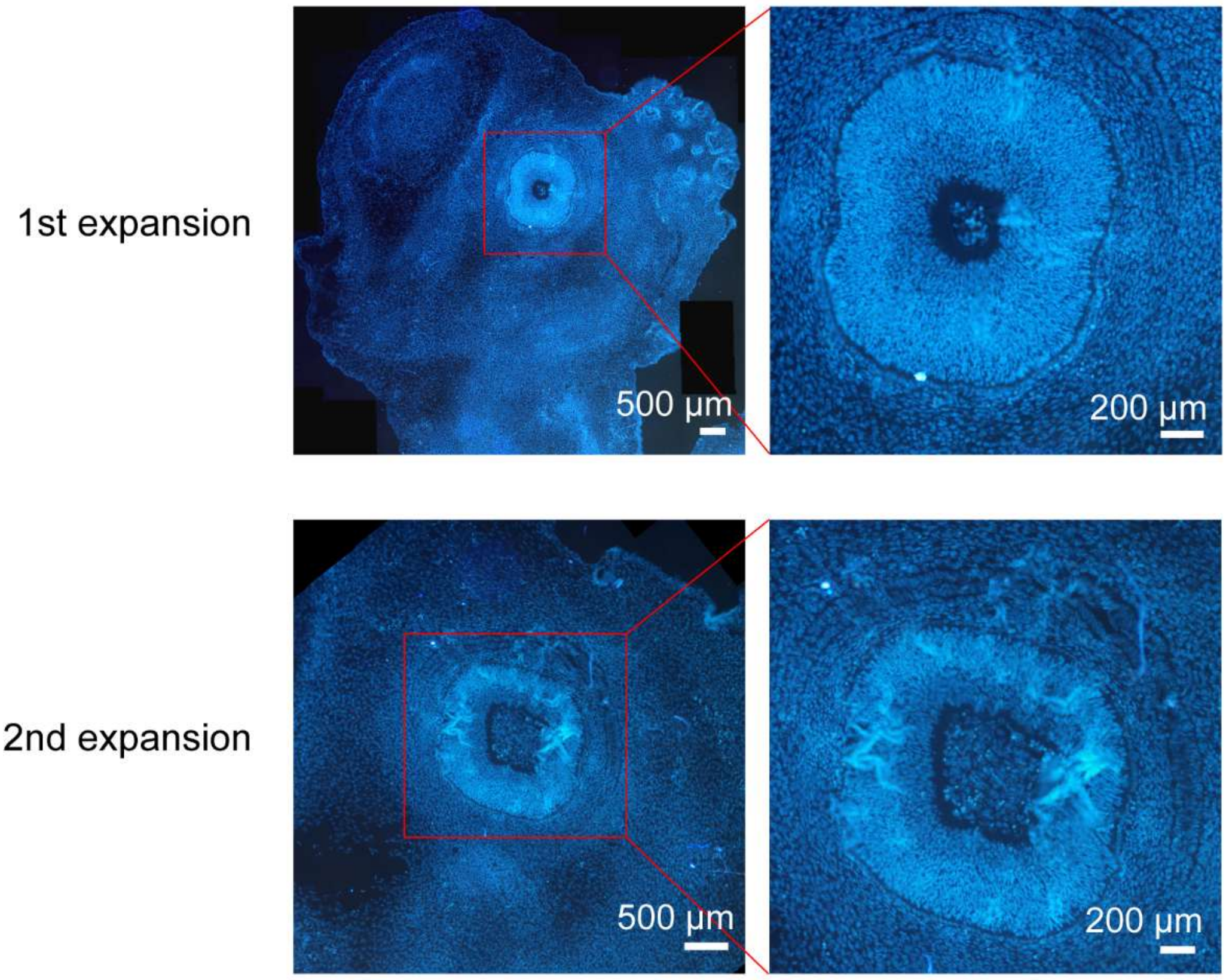

Figure S7. Representative DAPI images of the 1st- and 2nd-expansion samples after expansion, with magnified views showing local tissue morphology after expansion.

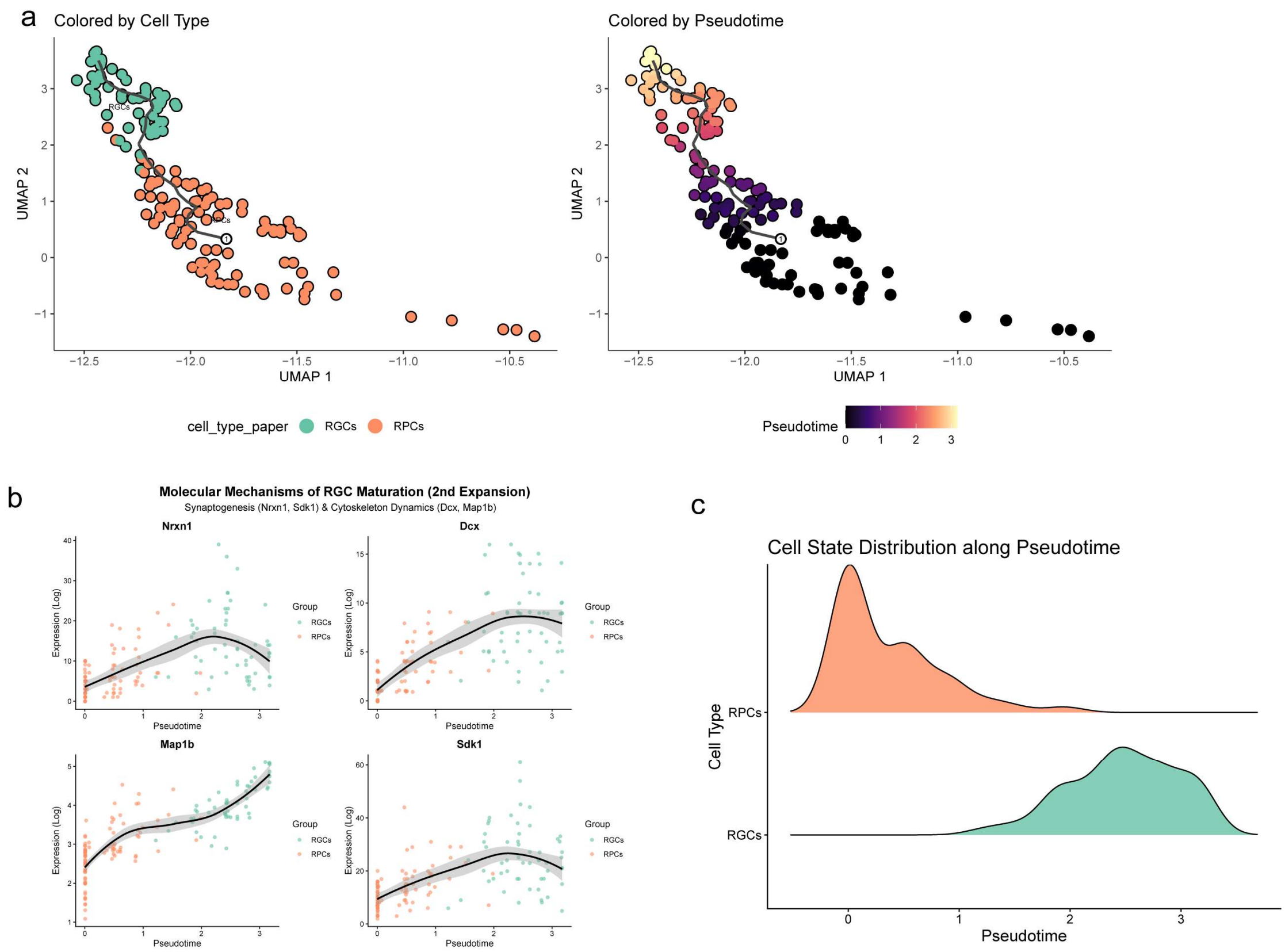

Figure S8. Pseudotime trajectory and molecular maturation in 2nd expanded retina. a) UMAP embedding of retinal spots colored by cell type (left) and pseudotime (right). b) Expression kinetics of maturation-associated genes (*Nrxn1*, *Sdk1*, *Dcx*, and *Map1b*) along the pseudotime axis using LOESS regression fits. c) Density distribution of RPC and RGC cell types along the pseudotime maturation timeline.

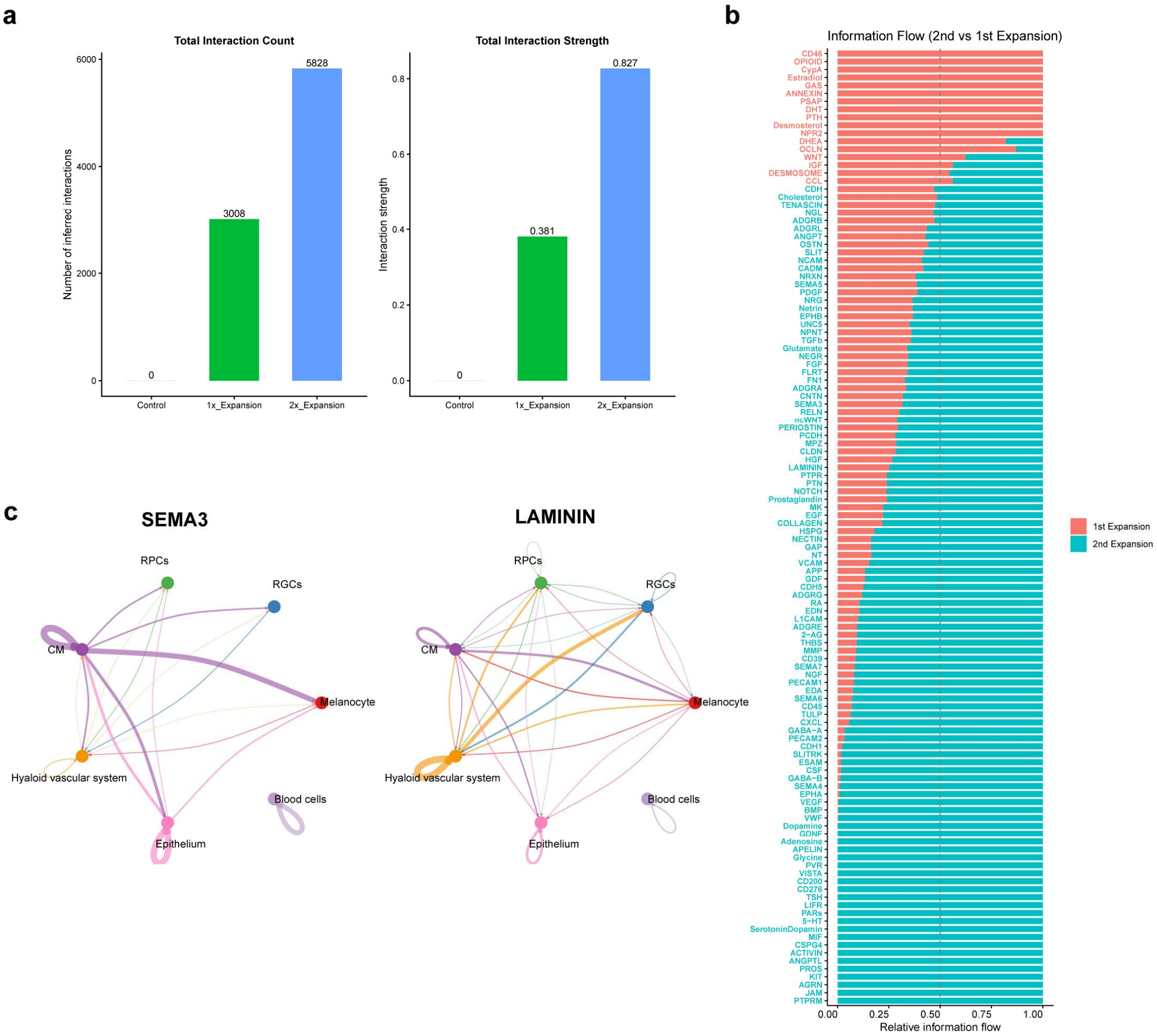

Figure S9. Expansion-enhanced sensitivity and global signaling networks. a) Total interaction counts and strengths across non-Expansion, 1st Expansion, and 2nd Expansion samples. b) Relative information flow analysis ranking signaling pathways by detection strength in 2nd versus 1st expanded samples. c) Network topology and spatial hierarchy of SEMA3 and LAMININ signaling across ocular clusters.

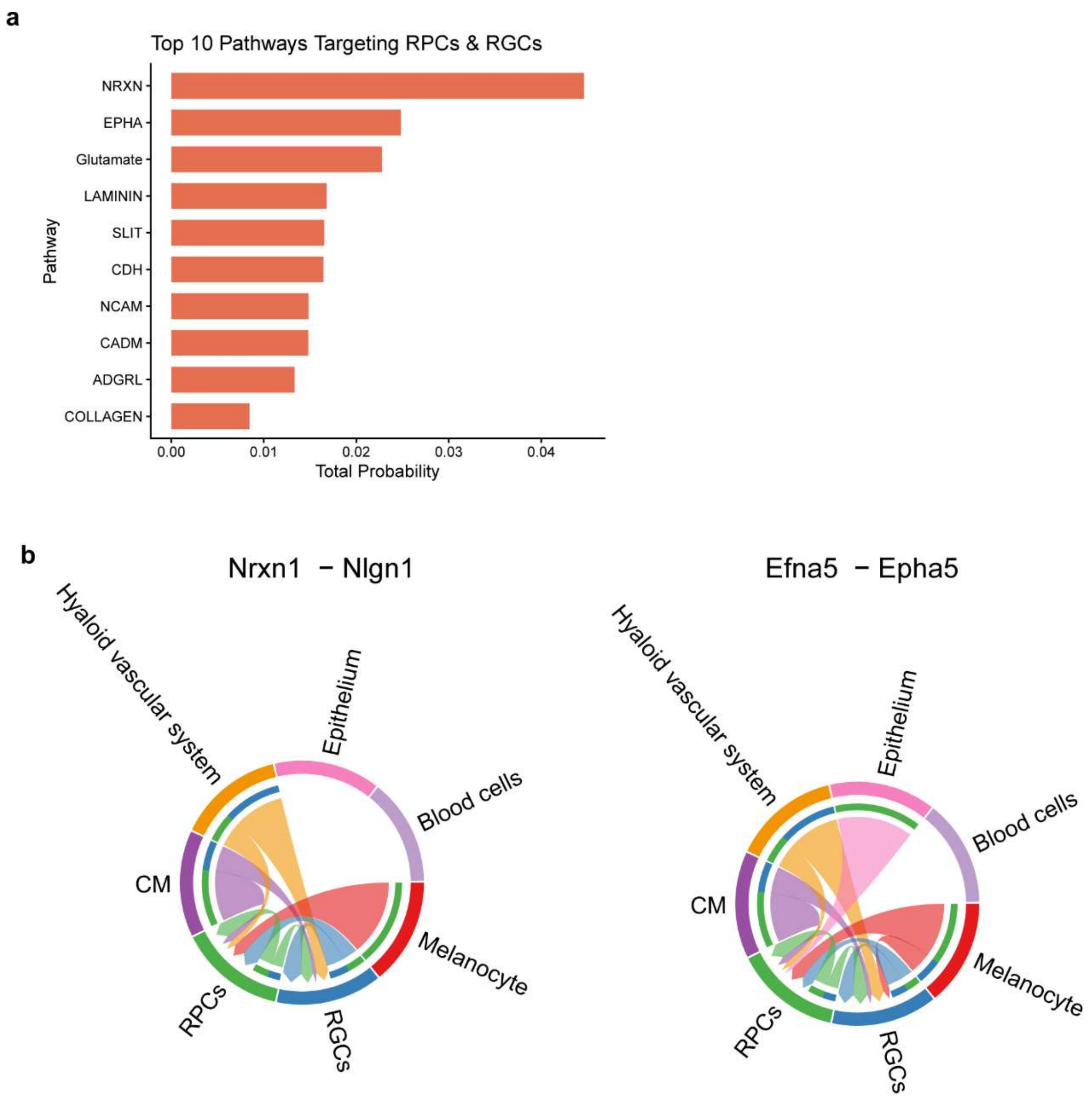

Figure S10. Targeted signaling niches for RPCs and RGCs. a) Ranking of the top 10 signaling pathways targeting RPC and RGC clusters based on total communication probability. b) Spatial communication networks and chord diagrams for *Nrxn1-Nlgn1* and *Efna5-Epha5* pairs.

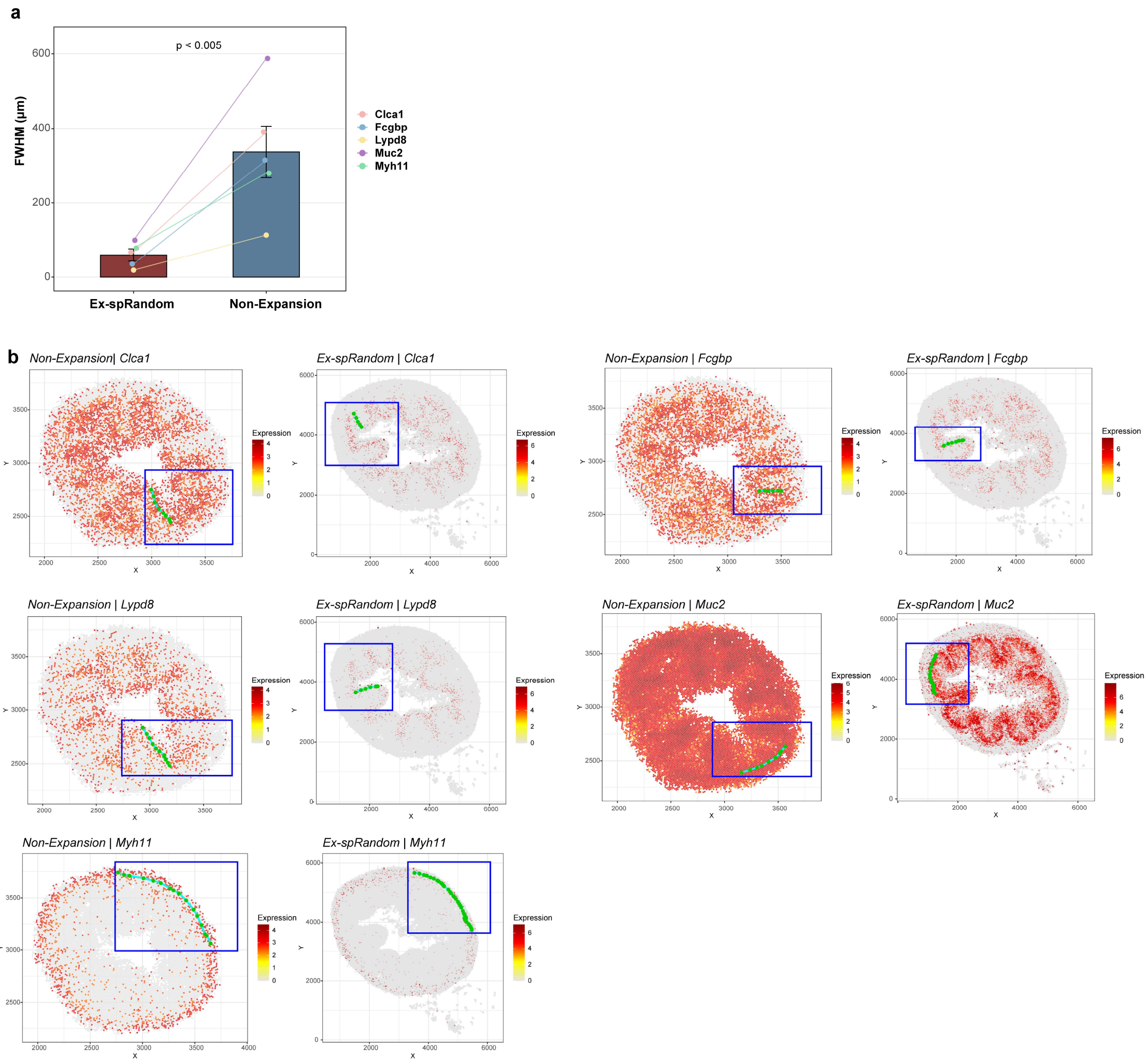

Figure S11. FWHM analysis of spatial expression profiles in the mouse colon. a, Comparison of FWHM values for *Clca1*, *Fcgbp*, *Lypd8*, *Muc2*, and *Myh11* between Ex-spRandom and non-expansion datasets generated using a 2.5-μm spatial barcode array. Colored points indicate individual genes, with pired measurements connected by lines. b, Spatial expression patterns of the indicated marker genes. Blue boxes indicate regions selected for analysis, and green lines indicate paths used for FWHM calculation. All measurements were converted to the original tissue scale.

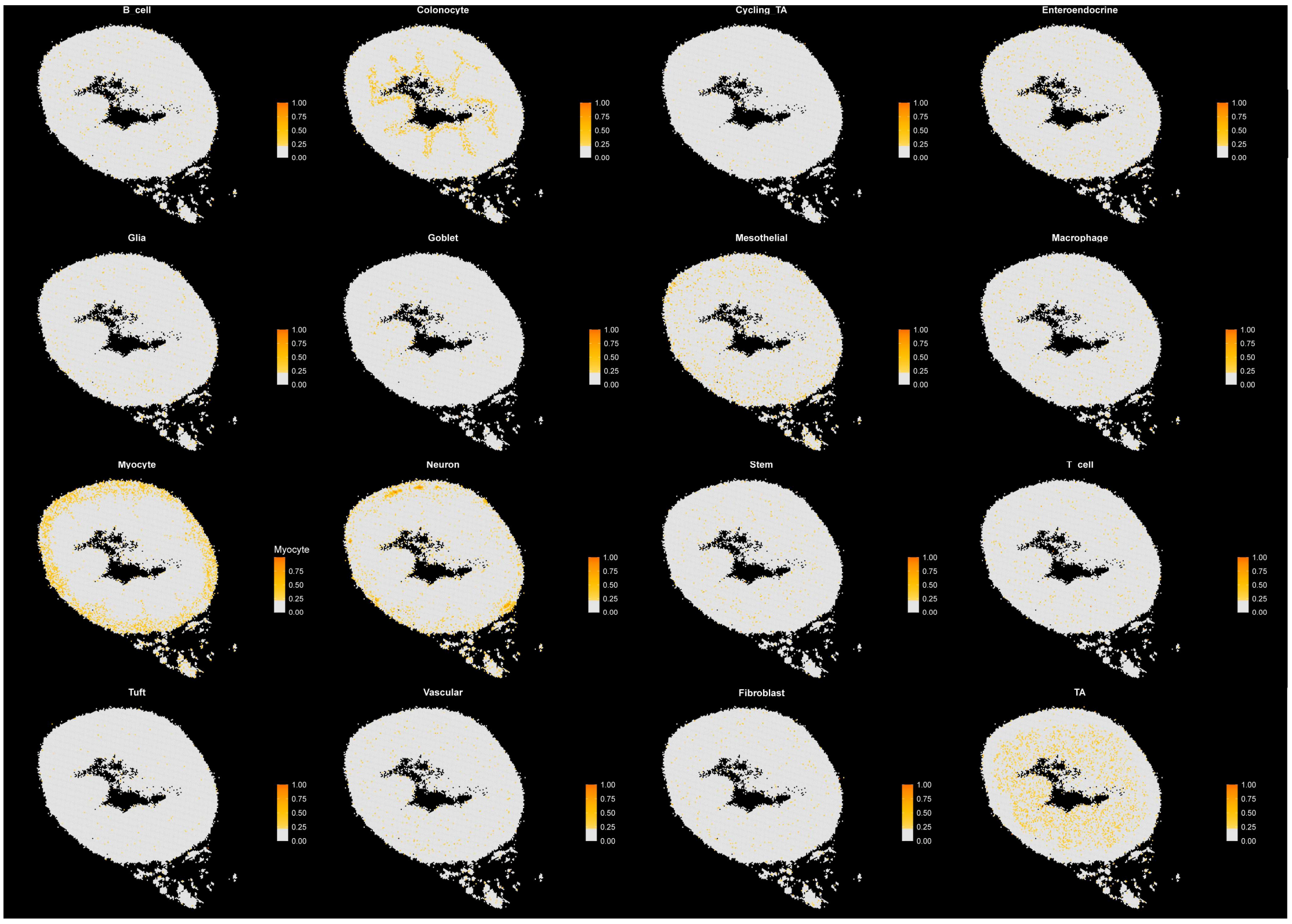

Figure S12. Spatial distribution of host cell types in the FFPE mouse colon. Spatial deconvolution maps generated by the RCTD algorithm showing the predicted probability densities of representative host cell populations, including colonocytes, goblet cells, transit-amplifying (TA and cycling TA) cells, enteroendocrine cells, neurons, and myocytes. The color scale (0.00 to 1.00) indicates the probability score of a specific cell type at each spatial spot.

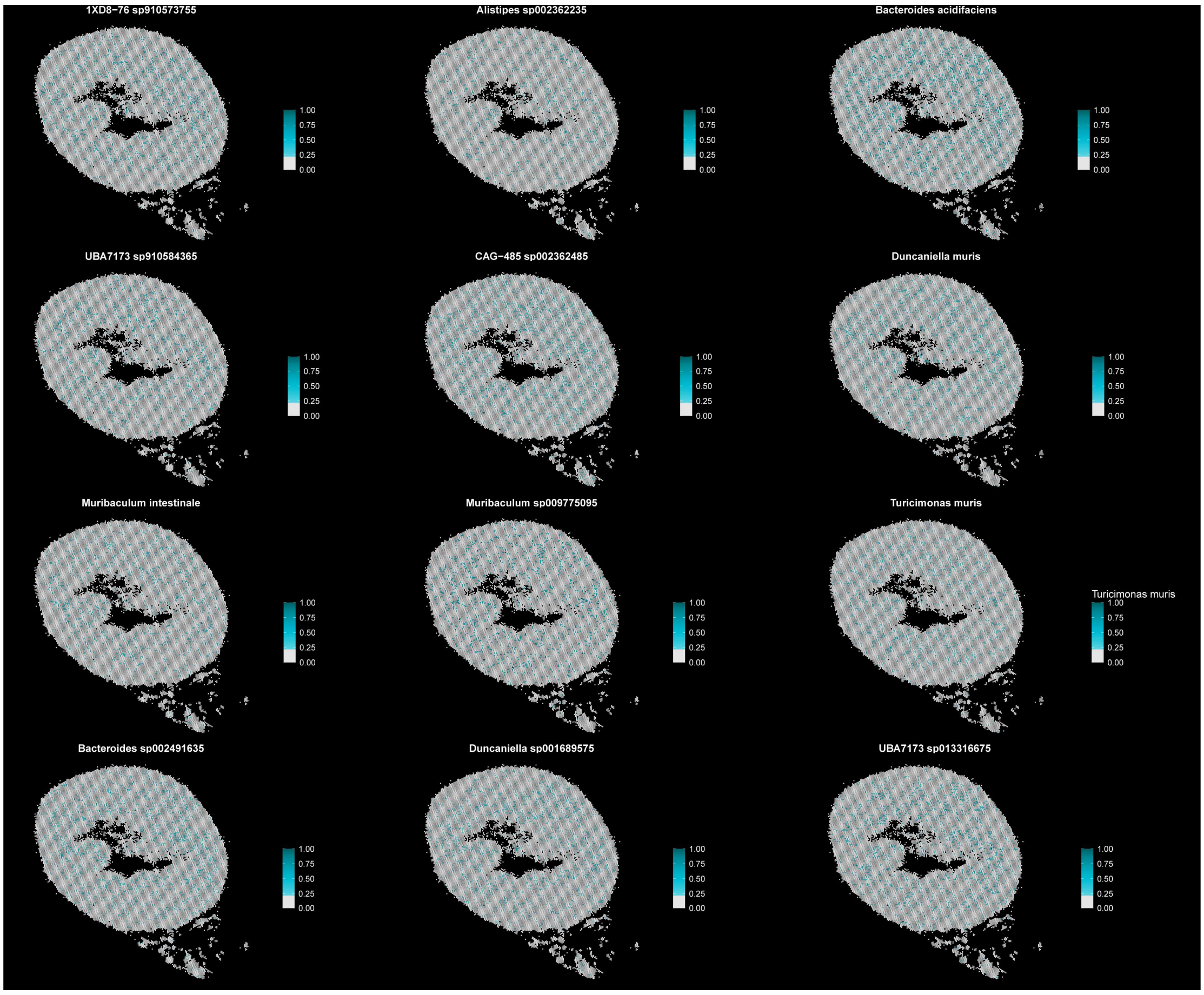

Figure S13. Spatial mapping of resident microbial taxa in the FFPE mouse colon. Spatial distribution maps of representative gut microbial taxa detected within the tissue section, including *Muribaculum intestinale*, *Bacteroides acidifaciens*, *Duncaniella muris*, *Alistipes*, and *Turicimonas muris*. The color scale (0.00 to 1.00) indicates the normalized relative abundance of each microbial species at corresponding spatial spots.

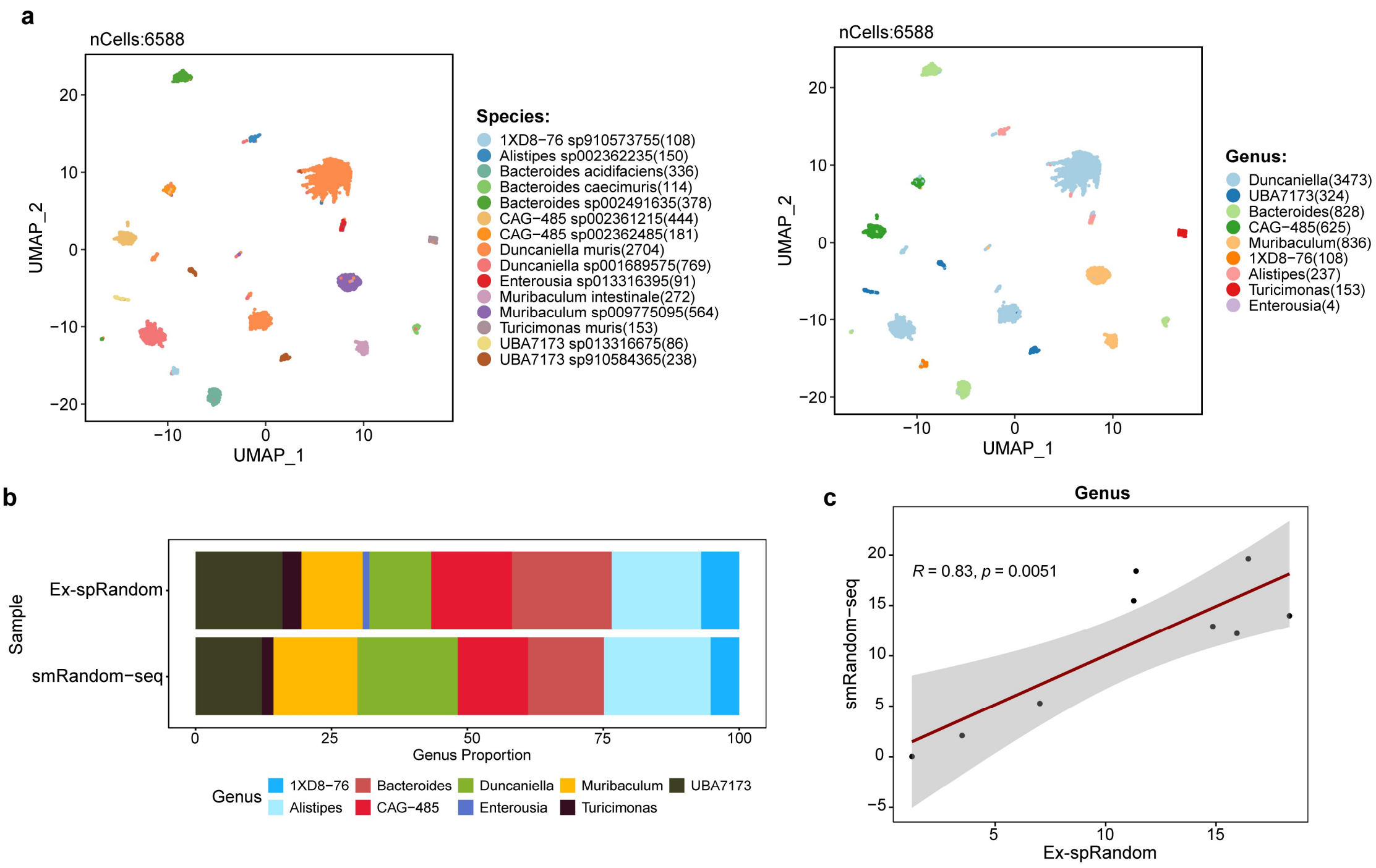

Figure S14. Cross-platform comparison of microbial taxonomic composition between Ex-spRandom and smRandom-seq. a, UMAP visualization of 6,588 microbial cells profiled by smRandom-seq and annotated at the species level (left) and genus level (right). b, Genus-level relative microbial composition detected by Ex-spRandom and smRandom-seq in the corresponding intestinal-content sample. c, Correlation of genus-level relative abundances between Ex-spRandom and smRandom-seq. Each point represents one genus; the red line indicates the linear regression fit and the gray band indicates the 95% confidence interval.

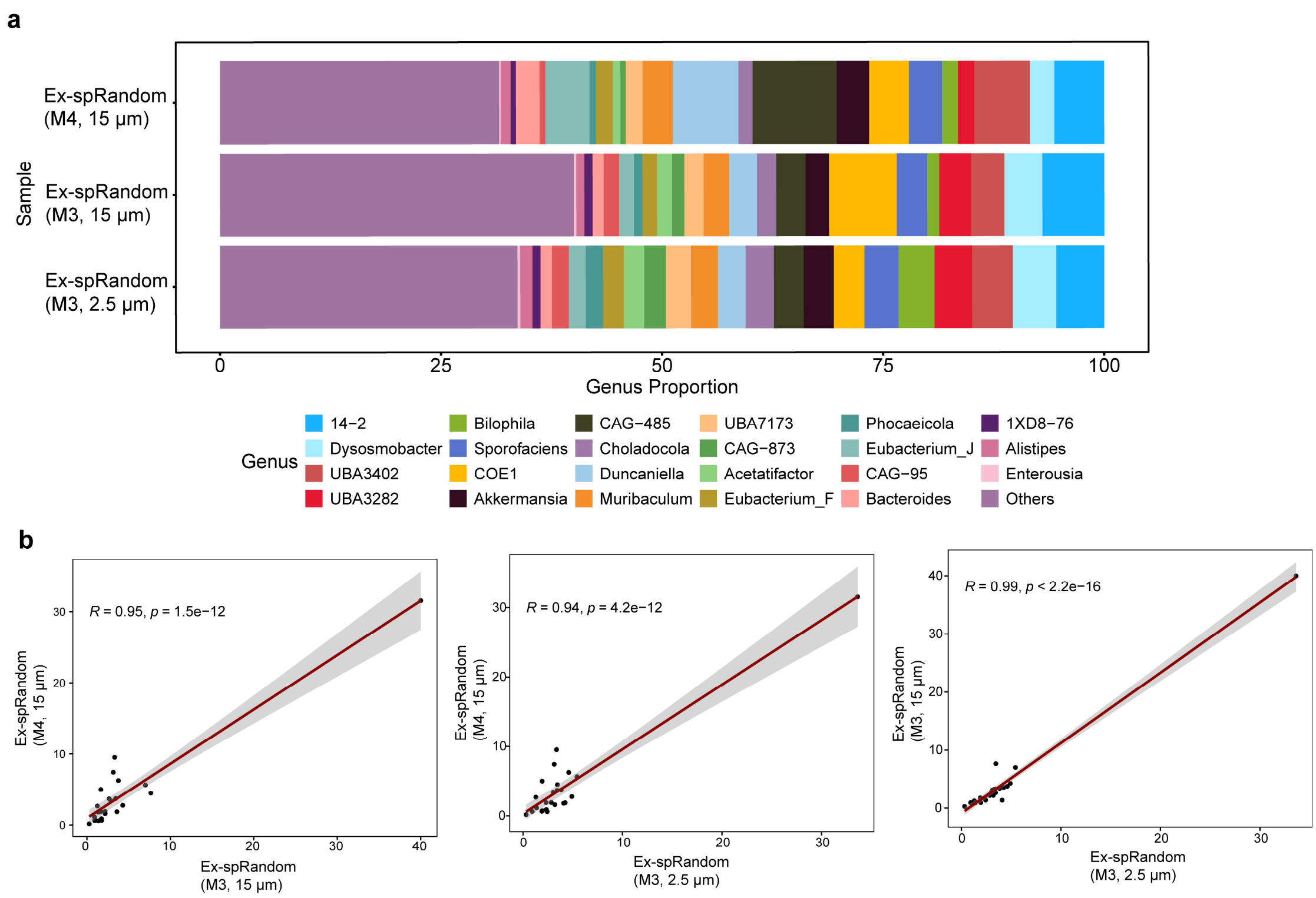

Figure S15. Genus-level microbial composition and pairwise concordance across Ex-spRandom colon datasets from independent mice and different spatial barcode arrays. a, Genus-level relative microbial composition in FFPE colon sections from two independent mice, M3 and M4. The 15-µm datasets from M3 and M4 were used for the matched cross-animal comparison, whereas the 2.5-µm dataset from M3 is shown as an additional dataset generated using a different barcode-array pitch. Genera not individually displayed are grouped as "Others." b, Pairwise correlations of genus-level relative abundances between M3 at 15 µm and M4 at 15 µm (left; Pearson's R = 0.95), M3 at 2.5 µm and M4 at 15 µm (middle; R = 0.94,), and M3 at 2.5 µm and M3 at 15 µm (right; R = 0.99). Each point represents one microbial genus. Red lines indicate linear regression fits, and gray bands indicate 95% confidence intervals. The values of 15 µm and 2.5 µm refer to the spatial barcode-array pitch.

References

1. Liao, Y. et al. Spatial Total RNA Sequencing of Formalin-Fixed Paraffin Embedded Tissues by spRandom-seq. *bioRxiv*, 2025.2002.2019.638981 (2025).
2. Bai, Z. et al. Spatially exploring RNA biology in archival formalin-fixed paraffin-embedded tissues. *Cell* **187**, 6760-6779.e6724 (2024).
3. Zhao, Y. et al. Stereo-seq V2: Spatial mapping of total RNA on FFPE sections with high resolution. *Cell* **188**, 6554-6571.e6521 (2025).

4. Alon, S. et al. Expansion sequencing: Spatially precise in situ transcriptomics in intact biological systems. *Science* **371**, eaax2656 (2021).
5. Wang, G., Moffitt, J.R. & Zhuang, X. Multiplexed imaging of high-density libraries of RNAs with MERFISH and expansion microscopy. *Scientific Reports* **8**, 4847 (2018).
6. Wang, Y. et al. EASI-FISH for thick tissue defines lateral hypothalamus spatio-molecular organization. *Cell* **184**, 6361-6377.e6324 (2021).
7. Fan, Y. et al. Expansion spatial transcriptomics. *Nature Methods* **20**, 1179-1182 (2023).
