# Supplementary material for: Highly Sensitive Spatial Host‐Microbiome Transcriptomics in FFPE Tissues via Iterative Hydrogel Expansion
Source: Adv Sci (Weinh). 2026 Aug 17:e77219. Online ahead of print. doi: 10.1002/advs.77219 (PMC13479583; doi:10.1002/advs.77219)
Supplement: Supplementary file 2 [file ADVS-9999-e77219-s001.docx]

**Highly sensitive spatial host-microbiome transcriptomics in FFPE tissues via iterative hydrogel expansion**

Shunji Zhang, Mengyuan Wang, Jiaye Chen, Yuyi Zhu, Bin Zhu, Ziye Xu, Yuan Liao*, Yongcheng Wang*

S. Zhang, M. Wang, J. Chen, Z. Xu, Y. Liao, Y. Wang

Department of Laboratory Medicine of The First Affiliated Hospital & Liangzhu Laboratory, Zhejiang University School of Medicine, Zhejiang University, Hangzhou, China

E-mail: (yongcheng@zju.edu.cn (Y.W.); ly62_2012@163.com (L.Y.))

S. Zhang, Y. Wang

College of Biomedical Engineering and Instrument Science, Zhejiang University, Hangzhou, China

Y. Zhu, B. Zhu

M20 Genomics, Hangzhou, China

Shunji Zhang, Mengyuan Wang and Jiaye Chen contributed equally to this work.

**Table of contents**

Ex-spRandom protocol…………………………………………………………….3

| **REAGENT OR RESOURCE** | **SOURCE** | **IDENTIFIER** |
| --- | --- | --- |
| Xylene | Aladdin | Cat# X112050 |
| Ethanol | Aladdin | Cat# E130059 |
| 20X SSC Buffer | Sangon | Cat# B548110-0200 |
| Tris EDTA Buffer pH 9 | Biorigin | Cat# BN24317 |
| Lysozyme | Thermo Fisher | Cat# 90082 |
| 100 mM dNTP | Thermo Fisher | Cat# 18427089 |
| 100mM dATP | NEB | Cat# N0440S |
| Reverse transcriptase and 5× RT buffer | M20 Genomics | Cat# EFT131-1-ER |
| Terminal transferase, terminal transferase reaction buffer pack and CoCl2 | NEB | Cat# M0315S |
| 10× ThermoPol buffer | NEB | Cat# B9004S |
| DNA polymerase | M20 Genomics | Cat# R20123124 |
| RNase H | NEB | Cat# M0297S |
| Murine RNase inhibitor | Vazyme | Cat# R301-03 |
| 8 M KOH | Sigma | Cat# P4494-50ML |
| 2X KAPA HiFi HotStart ReadyMix | Roche | Cat# KK2602 |
| SPRIselect beads | Beckman | Cat# B23318 |
| Triton X-100 | Sigma | Cat# 9002-93-1 |
| N,N,N',N'-tetramethylethylenediamine (TEMED) | Adamas | Cat# CC000530000-100mg |
| Sodium acrylate | Adamas | Cat# 100212C |
| N,N-Dimethylacrylamide (DMAA) | Adamas | Cat# 43340B |
| Ammonium persulfate (APS) | Sigma-Aldrich | Cat# A367825G |
| Acrylamide (AA) | Adamas | Cat# 83627N |
| N,N'-Methylenebisacrylamide (BIS) | Innochem | Cat# A16528 |
| M20 Genomics slides (15 and 50 μm) | M20 Genomics | N/A |
| BMKMANU S3000 (2.5um) | BMKMANU | Cat# STO3009 |

**Ex-spRandom protocol**

**FFPE sections treatment**

1. FFPE sections were cut from the paraffin block and were kept on at 60℃ for 2h.
2. The sections were then washed twice with 4 ml Xylene for 20 min at room temperature.
3. The sections were immersing in a graded series of ethanol solutions (100%, 100%, 90%, 70%, 50%, and 30%) for 3 min each time. The sections were washed three times with 4 ml pre-cold wash buffer (1X PBS with 1U/μL RNase inhibitor) at room temperature for 5 min.

**H&E staining and imaging**

1. The sections were then stained using standard Hematoxylin and Eosin (H&E) staining reagents, including hematoxylin staining for 5 min, bluing for 1 min , and eosin staining for 1 min, with Milli-Q water washes after each staining step.
2. The sections were then mounted with 80% glycerol and covered with coverslips.
3. Bright-field images were acquired using a Leica CS2 microscope with a ×10 objective.

**Permeabilization and decrosslinking**

The lysozyme permeabilization solution was prepared: 165.5 μL of 1× PBS, 20 μL of lysozyme buffer containing 1 M Tris-HCl and 0.5 M EDTA, 10 μL of lysozyme (50 mg/mL), 2 μL of 10% Triton X-100, and 2.5 μL of RNase inhibitor (20 U/μL), for a final volume of 200 μL.

1. The sections were treated with 200 µL of 0.1 M HCl at 42 °C for 15 min.
2. Then the sections were rinsed thoroughly with TE buffer (pH 9.0)
3. The FFPE sections were decrosslinking using pH 9.0 TE buffer, incubated at 95℃ for 60 min and then washed three times with 2X SSC buffer (2X SSC buffer with 1U/μL RNase inhibitor).
4. For FFPE sections containing bacteria, the sections were first washed with PBS containing 0.04% Tween-20 and RNase inhibitor. Bacterial cell-wall permeabilization was then performed using freshly prepared lysozyme permeabilization solution containing 2.5 mg/mL lysozyme and 0.1% Triton X-100. The sections were incubated at 37 °C for 30 min and then promptly washed three times with 2× SSC buffer containing RNase inhibitor. This step was performed only for FFPE sections containing bacteria.

**Reverse transcription**

The reverse transcription mix was prepared: 55 μL of ddH_2_O, 20 μL of 5X reverse transcription buffer, 5 μL of 100 mM dNTP, 10 μL of 10 μM random primer, 5μL of Murine RNase Inhibitor, 5μL of reverse transcriptase.

1. The FFPE sections were incubated with the 100 μL of reverse transcription mixture and subjected to eight cycles of ramped annealing: 8 °C for 1 min, 15 °C for 2 min, 20 °C for 3 min, 25 °C for 4 min, 30 °C for 4 min, and 42 °C for 8 min. Reverse transcription was then continued at 42 °C for 1 h, followed by holding at 4 °C overnight.
2. After incubation, the sections were washed with 2X SSCT three times to wash away the residual random primers and primer dimers.

**dA tailing**

The dA tailing mix was prepared: 78 μL of ddH_2_O, 10 μL of 10X TDT buffer, 10 μL of CoCl_2_ buffer, 1 μL of 100mM dATP, 1 μL of TdT enzyme.

1. The FFPE sections were incubated with the 100 μL of dA tailing mix at 37℃ for 30 min.
2. The sections were washed with 2X SSCT three times.

**FFPE Tissue Expansion**

The gel precursor solution (monomer1) was prepared: 332 μL of 100% sodium acrylate (SA), 100 μL of 100% acrylamide (AA), 5 μL of 1% N,N′-methylenebisacrylamide (BIS), 40 μL of N,N-dimethylacrylamide (DMAA), and ddH₂O to a final volume of 1,000 μL. APS and TEMED were omitted during the infiltration step.

1. For FFPE sections mounted on adhesive slides, a pre-freezing step was applied immediately before gel infiltration. The slides were placed at −80 °C for 15–30 min, briefly rinsed once with 1 mL PBS, incubated in 1 mL PBS at room temperature for 3 min, and then the PBS was completely removed. For FFPE sections mounted on non-adhesive slides, this pre-freezing step was omitted.
2. The tissue sections were incubated with 200 μL gel precursor solution (monomer1; without APS/TEMED) at 4 °C for 20 min to allow sufficient monomer infiltration.
3. After infiltration, the gel precursor solution was carefully removed. A 3-mm-high metal ring was placed around the tissue region, and a coverslip was gently positioned on top of the ring to form a confined gelation chamber.
4. Fully activated gel solution containing 0.1% APS and 0.1% TEMED was introduced from the side of the coverslip until the tissue was completely submerged.
5. The samples were incubated at 4 °C for 20 min to allow further equilibration and then polymerized at 37 °C for 40 min.
6. After polymerization, the coverslip was carefully removed, and the gel-embedded tissue was detached from the slide and transferred into 50 mL of 1× PBS.
7. The gel was allowed to expand at 4 °C until no further obvious size change was observed.
8. After physical expansion, excess gel surrounding the tissue was mechanically trimmed, and only the region of interest was retained for downstream spatial capture.

**Secondary Expansion(optional)**

The stability-gel monomer solution (monomer2) was prepared: 100 μL of 40% acrylamide/bis-acrylamide solution (AA:BIS, 19:1) and 900 μL of ddH₂O, yielding a final 4% AA:BIS monomer solution. APS and TEMED were omitted during the infiltration step.

1. Tissue-containing gels that had already undergone the first expansion step were incubated in 200 μL of stability-gel monomer solution (monomer2; without APS/TEMED) at 4 °C for 20 min.
2. The monomer2 solution was removed and replaced with 200 μL of activated monomer2 containing 0.1% APS and 0.1% TEMED.
3. The samples were incubated at 4 °C for 20 min and polymerized at 37 °C for 20 min.
4. After polymerization, excess peripheral monomer2 gel was mechanically trimmed away.
5. The stabilized gels were then incubated in 200 μL of expansion-gel monomer solution (monomer1; without APS/TEMED) at 4 °C for 20 min.
6. After monomer1 infiltration, the tissue-facing side of the gel was placed tightly against a coverslip. Vacuum grease was applied to the four corners of the coverslip to stabilize the assembly.
7. The coverslip–gel assembly was inverted onto a glass slide so that the non-tissue side of the gel was also in close contact with the slide surface.
8. 200 μL of activated monomer1 containing 0.1% APS and 0.1% TEMED was introduced into the assembly.
9. The samples were incubated at 4 °C for 20 min and polymerized at 37 °C for 40 min.
10. After polymerization, excess gel was mechanically trimmed. The samples were then immersed in 1× PBS and allowed to expand until no further obvious size change was observed.
11. The secondarily expanded gels were used for downstream cDNA transfer.

**cDNA Transfer from Expanded Gel to Spatial Barcode Chip**

The cDNA transfer solution was prepared: 11.85 μL of ddH₂O, 0.75 μL of 10% Triton X-100, 0.9 μL of RNase H, and 1.5 μL of 10× ThermoPol buffer, for a final volume of 15 μL.

1. After tissue expansion and gel trimming, the non-tissue side of the expanded gel was securely mounted against a coverslip to provide mechanical support during transfer.
2. A small amount of vacuum grease was applied around the perimeter of the spatially barcoded chip to stabilize the assembly and prevent leakage of the transfer solution.
3. 15 μL cDNA transfer solution was added directly onto the tissue-facing surface of the expanded hydrogel.
4. The gel was carefully placed onto the spatially barcoded chip, with the tissue-facing side in direct contact with the capture array.
5. Gentle and continuous pressure was applied through the supporting coverslip to ensure flat and uniform contact between the expanded tissue-gel surface and the chip.
6. The cDNA transfer reaction was performed at 37 °C for 30 min.
7. After transfer, the gel was carefully removed from the chip, and the chip was processed immediately for downstream extension, washing, and library preparation.

**DNA extension in situ**

The extension mix was prepared: 72 μL ddH_2_O, 10 μL 10× ThermoPol buffer, 10 μL 10mM dNTP and 8 μL DNA Polymerase.

1. The gene expression slides were incubated with the 100 μL of extension mix at 37℃ 30 min, 60℃ 60 min.
2. The gene expression slides were washed with 2X SSCT three times.

**Library preparation**

1. Following DNA extension, the standard 10X Visium CytAssist library preparation was followed to generate cDNA and final sequencing libraries: The single cDNA containing the spatial barcode was eluted using 50 μl 0.08 M KOH and 3 μl 1 M Tris-HCl pH 8.0.
2. Amplify the mix using 10X Genomics pre-amplification mix for 10 cycles and cleanup by SPRIselect. The cycles was: 98℃ 3 min; 10/rest cycles of 98℃ 15 s, 63℃ 20 s, 72℃ 30 s; End cycles, 72℃ 1 min; 4℃ forever.
3. The rest cycle number of determinations was performed by qPCR and final sequencing libraries was amplified by dual index primers. The libraries were then pooled and sequenced using the NovaSeq X (Illumina).
